# Predicting tumor response to drugs based on gene-expression biomarkers of sensitivity learned from cancer cell lines

**DOI:** 10.1186/s12864-021-07581-7

**Published:** 2021-04-15

**Authors:** Yuanyuan Li, David M. Umbach, Juno M. Krahn, Igor Shats, Xiaoling Li, Leping Li

**Affiliations:** 1 Biostatistics and Computational Biology Branch, National Institute of Environmental Health Sciences, 111 T.W. Alexander Dr., Research Triangle Park, MD A3-03, Durham, NC 27709 USA; 2 Genome Integrity & Structural Biology Laboratory, Research Triangle Park, Durham, NC 27709 USA; 3 Signal Transduction Laboratory, National Institute of Environmental Health Sciences, Research Triangle Park, Durham, NC 27709 USA

**Keywords:** Drug sensitivity, RNA-seq, Cancer cell line, GDSC, GA/KNN, TCGA, GTEx, And CCLE

## Abstract

**Background:**

Human cancer cell line profiling and drug sensitivity studies provide valuable information about the therapeutic potential of drugs and their possible mechanisms of action. The goal of those studies is to translate the findings from in vitro studies of cancer cell lines into in vivo therapeutic relevance and, eventually, patients’ care. Tremendous progress has been made.

**Results:**

In this work, we built predictive models for 453 drugs using data on gene expression and drug sensitivity (IC_50_) from cancer cell lines. We identified many known drug-gene interactions and uncovered several potentially novel drug-gene associations. Importantly, we further applied these predictive models to ~ 17,000 bulk RNA-seq samples from The Cancer Genome Atlas (TCGA) and the Genotype-Tissue Expression (GTEx) database to predict drug sensitivity for both normal and tumor tissues. We created a web site for users to visualize and download our predicted data (https://manticore.niehs.nih.gov/cancerRxTissue). Using trametinib as an example, we showed that our approach can faithfully recapitulate the known tumor specificity of the drug.

**Conclusions:**

We demonstrated that our approach can predict drugs that 1) are tumor-type specific; 2) elicit higher sensitivity from tumor compared to corresponding normal tissue; 3) elicit differential sensitivity across breast cancer subtypes. If validated, our prediction could have relevance for preclinical drug testing and in phase I clinical design.

**Supplementary Information:**

The online version contains supplementary material available at 10.1186/s12864-021-07581-7.

## Background

Studies that characterize human cancer cell lines and evaluate their sensitivity to drugs provide valuable information about the therapeutic potential and the possible mechanisms of action of those drugs. Those studies allow the identification of genomic features that are predictive of drug responses and make it possible to relate findings from cell lines to tissue samples and, ultimately, to translate laboratory results into patients’ care.

The Genomics of Drug Sensitivity in Cancer (GDSC) Project has assayed the sensitivity of 987 cancer cell lines to 320 compounds in their phase 1 (GDSC1) assay and of an additional 809 cancer cell lines to 175 compounds (some of which were included in the GDSC1 assay) in their phase 2 (GDSC2) assay [1–3]. The sensitivity of each cancer cell line to the drugs was represented as an IC_50_ value (the concentration at which a cell line exhibited an absolute inhibition in growth of 50%; lower IC_50_ implies higher sensitivity). GDSC also quantified the basal level gene expression of many of the cancer cell lines using microarray [1]. Concomitantly, other consortia such as the CCLE (cancer cell line encyclopedia) also profiled genome-wide gene expression of many of the cancer cell lines using RNA-seq [4, 5]. Additional genomic features such as somatic mutation and copy number variation, DNA methylation, epigenetic modifications, microRNA expression, and protein expression were also characterized by CCLE and others [4, 5]. The Cancer Therapeutics Response Portal (CTRP) project profiled the sensitivity of 860 cancer cell lines to 481 small molecules [6, 7]. The National Cancer Institute (NCI) has carried out a screening assay for a large number of small molecule compounds to detect potential anticancer activity using a group of 60 human cancer cell lines (NCI60) [8]. Recently, the transcriptomes of the NCI-60 cancer cell lines were also analyzed using RNA-seq [9]. Those resources make it possible to associate sensitivity of cancer cells to different drugs with genomic information on the cells, thereby, facilitating the discovery of molecular biomarkers of sensitivity and the identification genomic and genetic features that are predictive of cell sensitivity [5, 8–10].

GDSC applied various statistical and computational methods including elastic net regression and machine learning algorithms to identify multiple interacting genomic features influencing each cell line’s sensitivity to drugs. These analyses identified many interactions between cancer gene mutations and specific drugs [3]. For example, cancer cell lines with mutations in the BRAF genes are significantly more sensitive to PLX4730, a BRAF-inhibitor, than those with wild-type BRAF [3]. Additional computational methods for predicting sensitivity to drugs using gene expression data from cancer cell lines have been developed, for example [11–14]. Several publications have recent efforts in this area [15–18].

In addition to efforts directed at understanding the relationship between drug sensitivity and the genomic and genetic characteristics of cancer cell lines, major efforts have been put into relating the findings from in vitro studies of cancer cell lines to in vivo relevance. For example, Iorio et al. [2] carried out a comprehensive characterization of genomic alternations including somatic mutations, copy number alterations, and DNA methylation in 11,289 tumors and 1001 cancer cell lines. Tumor sensitivity to 265 drugs was predicted using corresponding sensitivity data from cancer cell lines by mapping cancer-driven alterations - the cancer functional events (CFEs) - in the tumors to cancer cell lines [2]. The authors identified single CFEs or combinations of them as markers of response and used a deep learning method to identify associations between drug molecular descriptors and mutational fingerprints in cancer cell lines. They subsequently used such associations to predict the potential of repurposing FDA approved drugs for cancer treatment [19]. Similarly, DeepDR considered genomic profiles of both cancer cell lines and tumors to predict tumor sensitivity to drugs using a deep neural network [20].

Those genomic-based approaches uncovered oncogenic alterations that are susceptible to anti-cancer drugs, thereby helping to identify treatment options that are tethered to specific genetic aberrations.

Conversely, other approaches relate cell-line findings to tumors based on transcriptome data. For example, using expression and drug sensitivity data from cancer cell lines, Geeleher et al. [21, 22] developed gene expression-based models to predict sensitivity to drugs; they subsequently applied the models to gene-expression data from TCGA tumor samples to impute sensitivity of the tumor samples to 138 drugs.

Similarly, we sought to identify gene expression signatures from cancer cell lines that can predict their sensitivity to drugs; and, subsequently, we used those signatures to predict the sensitivity of normal and tumor tissue to the drugs. Our work, however, differs from the previous work in several ways: a) our analysis is more comprehensive by including the latest drug sensitivity data from GDSC2 for 453 drugs; b) our work emphasizes identification of putative biomarkers of sensitivity to drugs and potential therapeutic options for cancer subpopulations; and c) we also predict toxicity of drugs to normal tissues using transcriptomic data from normal human tissues available from both The Cancer Genome Atlas (TCGA) and The Genotype-Tissue Expression (GTEx) project.

We identified many known drug-gene interactions and uncovered several potentially novel drug-gene associations. We predicted that OSI-027 (mTOR inhibitor) is a breast cancer specific drug with high specificity for the Her2-positive subtype breast tumors. Our analysis also suggests that *MULC1* expression is a surrogate marker for tumor response to OSI-027. Our analysis rediscovered the interaction between bleomycin and ACE (angiotensin I converting enzyme) [23]. We also predicted that other drugs are potentially specific for cancer (sub)types. Our prediction, if validated, could have relevance for preclinical drug testing and in phase I clinical design.

## Results

**Fig. 1:**
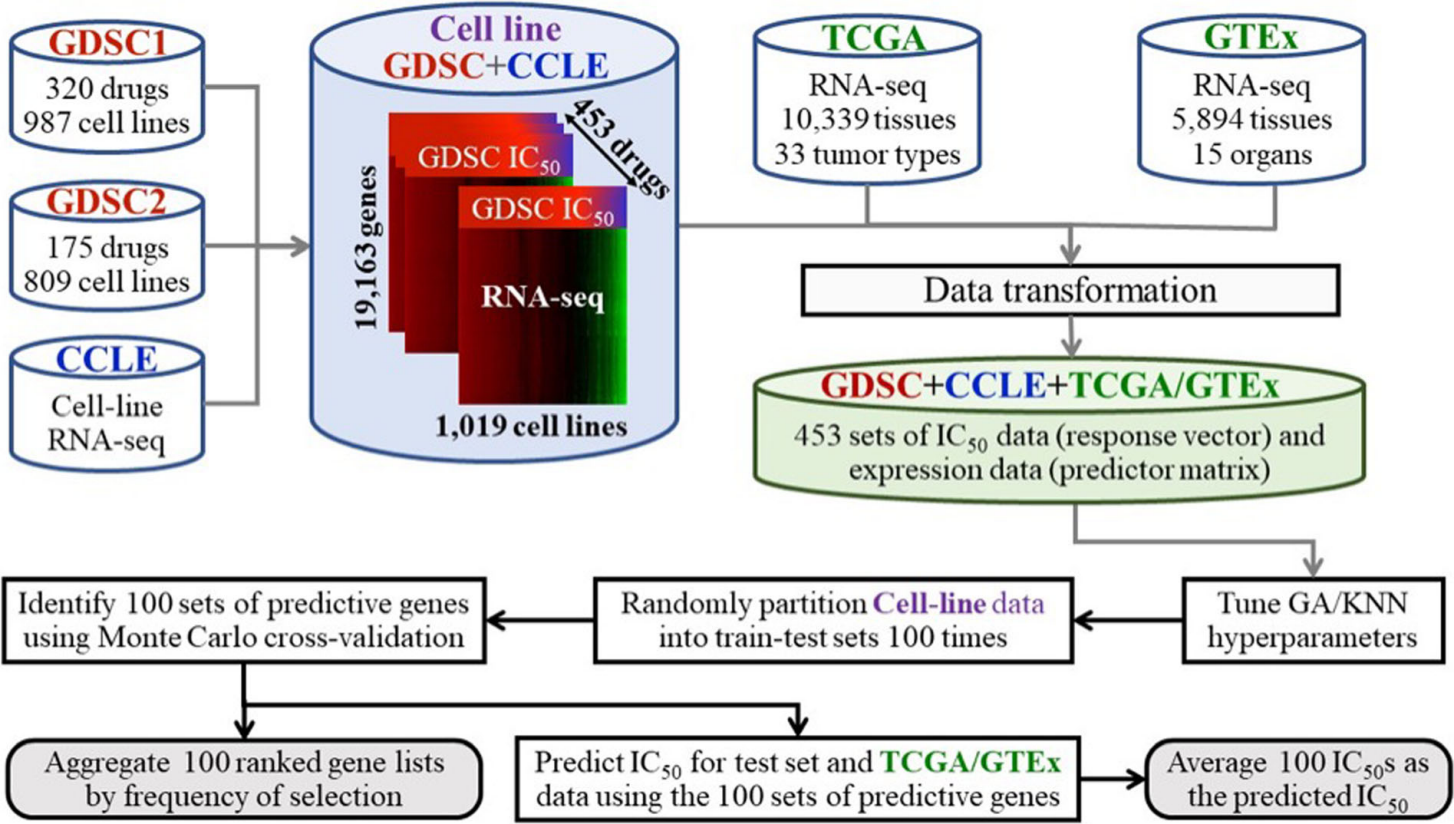
Schematic diagram of the work-flow. First, GDSC cancer cell line drug sensitivity data, CCLE cancer cell gene expression data and TCGA/GTEx tissue gene expression data are combined and transformed. The CCLE gene expression data and GDSC drug sensitivity data (collectively referred to as the cell-line data) were used to build predictive models that were subsequently used to predict/impute the tissue drug sensitivity for the TCGA and GTEx samples. Broadly, for each drug, we divided the cell-line data into a training and testing set. We aimed to identify a 30-gene set whose gene expression levels are most predictive of the IC_50_ values of the drug for the samples in the testing set. The resulting model (a 30-gene set) was subsequently used to predict the IC_50_ value of the TCGA/GTEx samples. This process was repeated 100 times independently. The predicted IC_50_ values from the 100 runs were then averaged and taken as the predicted IC_50_ value of the drug for the samples. For details, see Methods

Using cancer cell line gene expression data and cancer cell line drug sensitivity data, we built predictive models that were subsequently used to impute/predict tissue drug sensitivity using gene expression data for the tissues (Fig. 1). Details are provided in Methods.

### Training and testing performance for the cell-line data

First, we divided the cancer cell-line data into a training and testing set. We predicted the IC_50_ values of the cancer cell lines in the testing set for each of the 453 drugs. We computed both the Pearson (*ρ*_*P*_) and Spearman (*ρ*_*s*_) correlation coefficients between the observed and predicted IC_50_ values for the samples in the testing set. The median *ρ*_*P*_ and *ρ*_*s*_ coefficients between the observed and predicted IC_50_ values were 0.466 and 0.437 (Table 1), indicating that the basal transcriptomes of the cancer cell lines can reasonably predict the sensitivity (IC_50_s) of the cell lines to most of the drugs. Of the 453 drugs, 272 (60%) had both *ρ*_*P*_ and *ρ*_*s*_ testing-set correlations ≥0.4. We refer to those drugs as predictable drugs.

**Table 1:** Summary statistics of correlations between the observed and predicted ln (IC_50_) of the 453 drugs in the test set

Correlation	Min.	1st Qu.	Median	Mean	3rd Qu.	Max.
*ρ*_*P*_	−0.1990	0.3710	0.4660	0.4573	0.5510	0.7660
*ρ*_*s*_	−0.1800	0.3580	0.4370	0.4267	0.5070	0.6800

Interestingly, for 34 (7.5%) of the drugs, the cancer cell lines’ transcriptomes had little or no predictive power for the cell lines’ sensitivities to the drug (either *ρ*_*P*_ or *ρ*_*s*_ testing-set correlation coefficients ≤0.25). Moreover, we also confirmed that other transcriptomic data such as microRNA expression, DNA methylation, and protein expression (from reverse phase protein array) from CCLE and GDSC [4, 5] also had little or no predictive power for those drugs (data not shown). It is unclear why the transcriptomes of cancer cell lines failed to predict their sensitivity to those drugs. Some of 34 drugs had fewer than 100 samples with both gene expression and IC_50_ data and the lack of data may have contributed to those drugs’ poor prediction performance; for most of the others, however, data availability was not an issue.

For the remaining analyses, we focus on the top 272 predictable drugs – those having the highest testing-set correlations between the observed and predicted IC_50_ values (both *ρ*_*P*_ ≥ 0.4 and *ρ*_*s*_ ≥ 0.4). The top 10 predictable drugs (additional file 1: Table S1**)** appear to have diverse mechanisms of action.

### Top-ranked genes predictive of drug sensitivity

For each of the top 272 predictable drugs, we counted how many times each gene was selected into the 100 sets of the *d* (*d* = 30) predictive genes. For a transcriptome of 19,163 genes, a gene is expected by chance to be selected only 0.155 times [(30/19163) × 100] into 100 sets of 30 genes. We observed that many genes were being selected at frequencies more than 100 times above that expected by chance. The most frequently selected genes for each drug are potentially informative about that drug’s mechanism of action as well as about a cancer cell line’s sensitivity to the drugs. For some other drugs, multiple genes were selected with lower but distinctly higher-than-random frequencies, suggesting that multiple genes together are necessary for predicting cell-line sensitivity for those drugs. Many of the drug-gene interactions were also identified by others [1, 2, 5]. Among the predictable drugs, the number of genes selected into more than 20 of 100 predictive gene sets (i.e., > 100-fold above chance) ranged from 1 to 17 (additional file 2: Table S2).

*C19orf33* (chromosome 19 open reading frame 33) was among the most frequently included predictive genes for the largest number of drugs, appearing in more than 20% of the predictive gene sets for 17 drugs (additional file 2: Table S2). The expression level of *C19orf33* in cancer cell lines was positively correlated (*ρ*_*s*_ > 0.3) with the IC_50_ values of more than 100 drugs for those cell lines (additional file 3: Table S3), suggesting that higher expression of *C19orf33* in cancer cell lines was positively associated with higher resistance of the cancer cell lines to the drugs. No other genes were correlated with the IC_50_ values of as many drugs as was *C19orf33*. Most of the positively correlated drugs are DNA synthesis inhibitors, microtubule assembly inhibitors, or cell cycle inhibitors. Interestingly, *C19orf33* expression in cancer cell lines showed a negative correlation with the IC_50_ values of the kinase (MEK, ERK, SRC) inhibitors for the cell lines (additional file 3: Table S3), suggesting that cancer cell lines with higher *C19orf33* expression are more sensitive to kinase inhibitors than those with lower *C19orf33* expression. *C19orf33* encodes two transcript variants (Immortalization up-regulated protein 1 and 2: IMUP-1 and IMUP-2); both were discovered and characterized in immortalized cells as being upregulated compared to senescent cells [24]. IMUP-1 and IMUP-2 are more frequently expressed in cancer cells compared to normal tissues [24–26]. Overexpression of IMUP-1 and IMPU-2 in normal fibroblasts induces neoplastic transformation [27]. Our data suggested that *C19orf33* expression may be a general biomarker for the sensitivity of cancer cell lines to many chemotherapeutic agents.

For 14 drugs of diverse mechanisms of action, *ABCB1* appeared in more than 20% of the predictive gene sets. *ABCB1* encodes ATP binding cassette subfamily B member 1, a member of the superfamily of ATP-binding cassette (ABC) transporters. ABCB1, also commonly known as MDR1, is an ATP-dependent drug efflux pump for xenobiotic compounds with broad substrate specificity [28]. Expression of *ABCB1* is responsible for decreased drug accumulation in multidrug-resistant cells and often mediates the development of resistance to anticancer drugs [28–30].

#### Drugs whose top-ranked predictive genes matched the drug targets

Many of the most frequently selected genes were also known targets of the drugs (additional file 2: Table S2). The drug nutlin-3a inhibits the interaction between p53 and MDM2, leading to activation of the p53 pathway [31]. Gratifyingly, *MDM2* was selected in nearly 100% of gene sets predicting sensitivity to nutlin-3a. Other known p53 known target genes (*CDKN1A* and *RPL22L1*) were also selected in nearly 90% of those predictive gene sets, suggesting that high expression of these genes identifies tumors with a wild-type p53, and thus responsive to a further p53 activation by nutlin-3a.

The drugs PD173074 and AZD4547 are two potent fibroblast growth factor receptor (FGFR) inhibitors [32]; *FGFR2* was the most frequently selected gene for predicting sensitivity to these two drugs. Venetoclax is known to target BLC2 protein [33]; *BCL2* was selected in almost 100% of the predictive gene sets for sensitivity to venetoclax. Tanespimycin (also known as 17-AAG) is a HSP90 inhibitor, and *NQO1* expression is inversely correlated with 17-AAG IC_50_s in cancer cell lines [34]. Similarly, we found that *NQO1* was selected in 100% in the predicted gene sets for tanespimycin sensitivity. *ADK* (adenosine kinase) was selected in 100% of gene sets predicting sensitivity to AICAR. AICAR is an analog of adenosine monophosphate (AMP) that is capable of stimulating AMP-dependent protein kinase (AMPK) activity [35]. AICAR prevents the production of the enzymes adenosine kinase (ADK) and adenosine deaminase (ADA) [36].

*SPRY2* (sprouty RTK signaling antagonist 2) was among the most frequently selected genes for predicting sensitivity to all six MEK1/2 inhibitors in GDSC datasets (CI-1040, PD0325901, refametinib, SCH772984, selumetinib, and trametinib) (additional file 2: Table S2). Sprouty specifically inhibits activation of MAPK/ERK in response to a wide range of trophic growth factors [37, 38]. SPRY2 expression in cancer cell lines was inversely correlated with the IC_50_ values of all MEK inhibitors for the cancer cell lines (additional file 4: Fig. S1) with *ρ*_*s*_ ranging from − 0.24 to − 0.43 (all *p*-values <4E-08), indicating that cancer cell lines with higher *SPRY2* expression are more sensitive to the MEK inhibitors. The other most frequently selected genes for MEK1/2, BRAF, and ERK1/2 kinase inhibitors were *ETV4* (ETS variant transcription factor 4) and *SPRY4* (additional file 2: Table S2). ETV4 is a downstream target of ERK signaling pathway. In a mouse model, ETV4 promotes prostate cancer metastasis in response to coactivation of PI3-kinase and Ras signaling pathways [39]. Our results suggest that expression levels of *SPRY2* and *ETV4* are likely indicative of the sensitivity of cancer cell lines to many MAP kinase inhibitors. Other examples of drugs whose most frequently selected genes matched the drug-target genes include venetoclax-*BCL2*, navitoclax-*BCL2*, daporinad-*NAMPT* and savolitinib-*MET* (additional file 2: Table S2).

#### Drugs whose top-ranked genes did not match the drug targets

Our analysis also identified predictive genes that did not match the drug targets or genes for drugs with unknown mechanism of action. Here we provide one such example. *TRPM4* (transient receptor potential melastatin 4) was selected in 100% of gene sets that predicted sensitivity to acetalax. On the other hand, *TRPM4* was selected in fewer than 5% of the sets for all other drugs, suggesting that the *TRPM4*-acetalax interaction is specific. The mechanism of action for acetalax is unknown. Recent studies suggested that TRPM4 may be implicated in regulating cancer cell migration and in the epithelial-to-mesenchymal transition [40, 41]. The authors showed that overexpression of *TRPM4* in prostate cancer cell lines increased Snail protein expression and reduced expression of E-cadherin [40]. *TRPM4* expression in the CCLE cancer cell lines was inversely correlated with the IC_50_ of acetalax in those cell lines (*ρ*_*s*_ = − 0.45, *p*-value < 2.2E-16) (additional file 4: Fig. S2), suggesting that cancer cell lines with higher *TRPM4* expression are more sensitive to acetalax. It remains unclear, however, if acetalax has any effect on cancer cell migration or EMT in vitro.

### Predicting in-vivo drug sensitivity based on in-vitro data: proof of concept

**Fig. 2:**
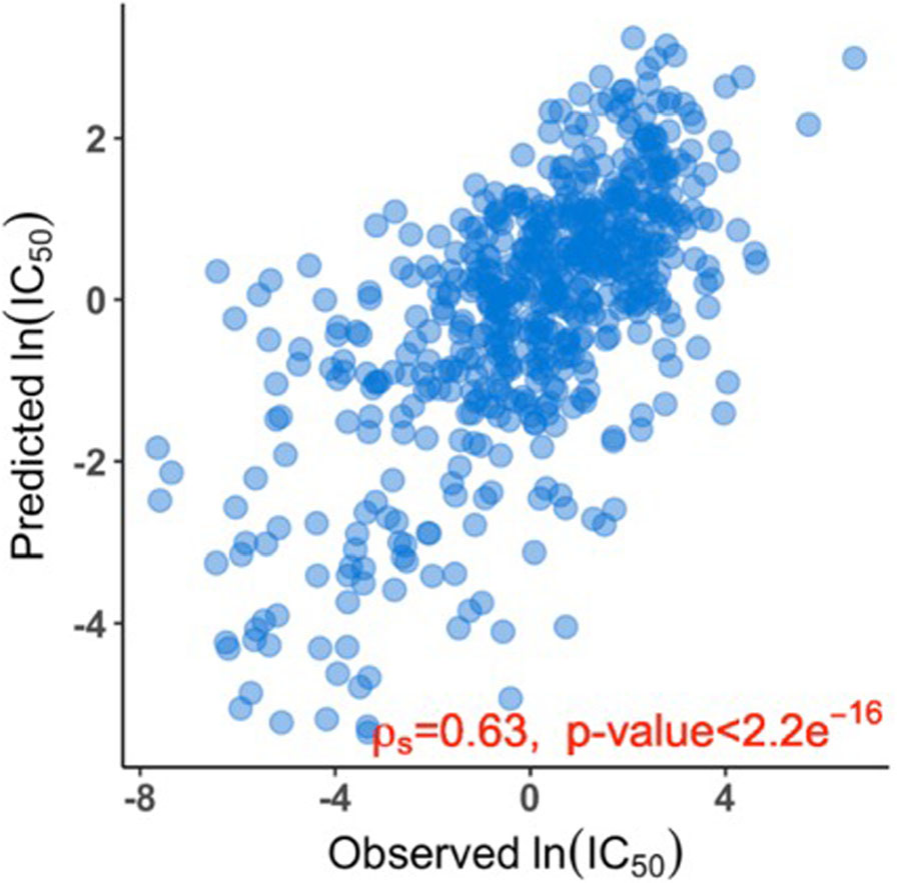
Scatter plot of predicted and observed ln (IC_50_) values for trametinib in the 571 cancer cell lines with both gene expression data and IC_50_ data for trametinib

If the transcriptome of a cancer cell line is predictive of that cell line’s sensitivity to a drug, we hypothesize that the transcriptome of a corresponding normal tissue is also predictive of that tissue’s sensitivity to the drug. To probe this hypothesis, we chose trametinib (GDSC drug ID = 1372, a MEK1/MEK2 inhibitor) [42] as an example. From the cell-line data, we observed that the ranks of the observed IC_50_ values of trametinib in the 571 cancer cell lines were associated with the corresponding IC_50_ values predicted by the cell-line gene expression levels (*ρ*_*s*_ = 0.635, *p*-value <1E-16) (Fig. 2). Trametinib specifically binds to and inhibits MEK1 and MEK2, resulting in an inhibition of growth factor-mediated cell signaling and cellular proliferation in various cancers [42]. Trametinib is clinically approved for stage IV as palliative treatment and for stage III as adjuvant treatment combined with BRAF inhibitors for BRAF+ melanoma [43–48]. GDSC assays also demonstrated that cancer cell lines from the skin and intestine are especially sensitive to trametinib [2] compared to those from other organs.

**Fig. 3:**
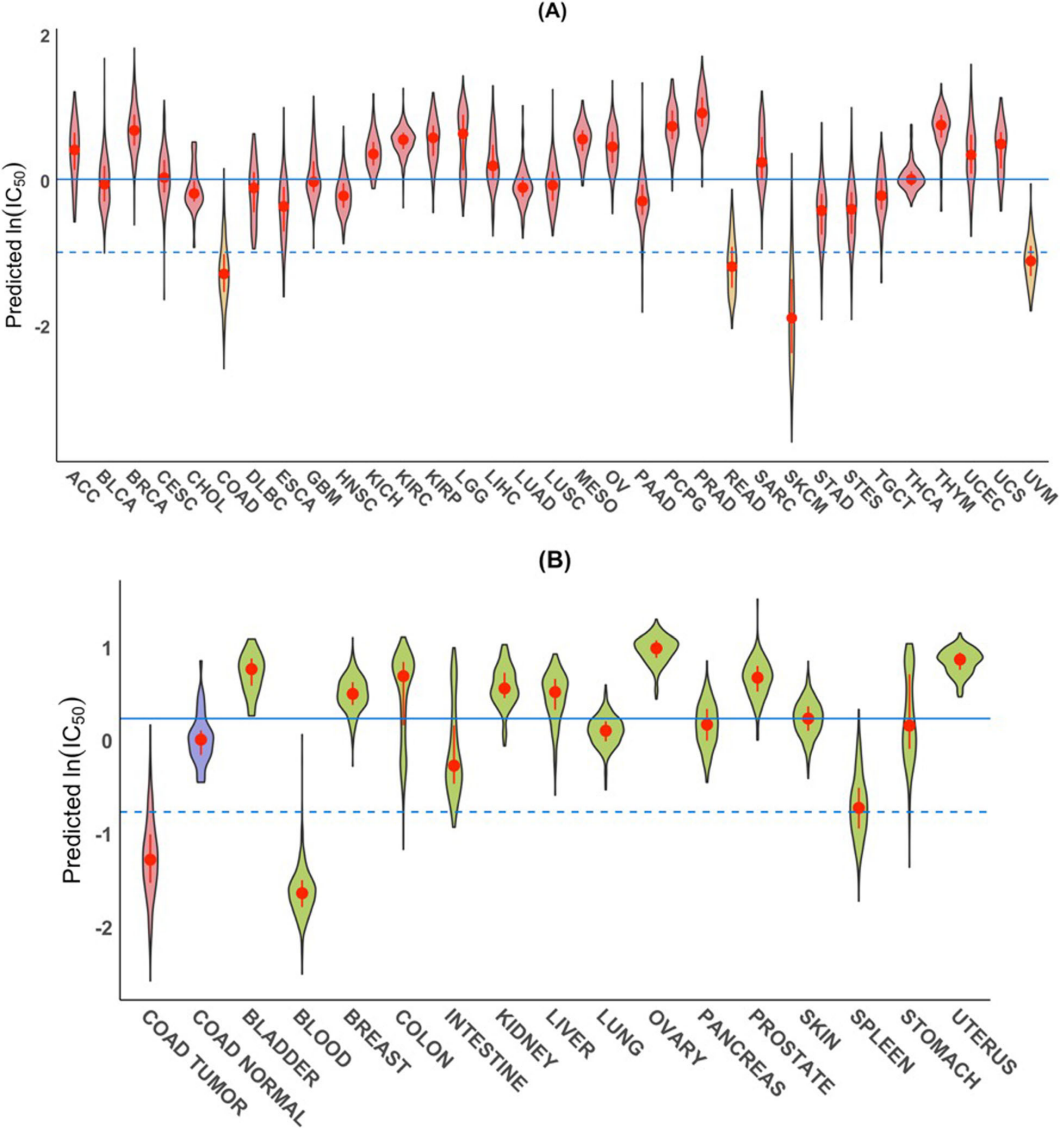
Predicted sensitivity of tumor-types and normal tissue to trametinib. **a**, Violin plots of predicted ln (IC_50_) values of trametinib based on RNA-seq gene expression data from TCGA tumor samples from 33 tumor types. Overall COAD, READ, SKCM and UVM tumors (yellow) had the lowest predicted median IC_50_ values. For the description of the 33 TCGA tumor types, see supplementary data (additional file 1: Table S7A). The solid line shows the median of the medians of the predicted IC_50_ values for all 33 tumor types whereas the dashed line is one logarithmic unit below the solid line. **b**, Violin plots of the predicted ln (IC_50_) values of trametinib for COAD tumor (red) and normal (blue) samples from TCGA and for GTEx normal tissue samples from 15 major organs (green); here the solid line shows the median of the medians of the predicted IC_50_ values for all 16 normal tissues. In each violin, the red dot is located at the median; the vertical red bar extends from 25th to 75th percentiles

Using the cell-line data as the training data, we predicted the IC_50_ values of trametinib for the ~ 11,000 TCGA RNA-seq samples. Our results indicated that the median predicted IC_50_ values of trametinib for melanoma (both skin and uveal) and intestine tumors (colorectal and rectal,) were much lower (showing higher sensitivity to trametinib) than those for all other tumor types (Fig. 3a). Those results are consistent with both trametinib’s specificity for cancer cell lines derived from those tissues [2] and its clinical efficacies; these consistencies suggest that our approach of linking in-vitro and in-vivo drug sensitivities is sound. Trametinib is effective in treating patients with colorectal tumors with BRAF V600E mutations [46, 47] and melanoma [49]. Moreover, our analysis further demonstrated that the median predicted IC_50_ values of trametinib are overall much lower for colorectal tumors than for the adjacent “normal” tissues (Fig. 3b). Likewise, our result indicated that trametinib is less cytotoxic to most normal organs except blood and spleen (Fig. 3b), both of which are hematopoietic related. Such tumor-to-normal selectivity is not common among the 272 drugs (additional file 5: Table S4; see below ‘Tumor-to-normal sensitivity’). It is also reassuring that the predicted IC_50_ values of trametinib for normal intestines from GTEx are comparable to those for the TCGA “normal” colon tissues (Fig. 3b). Interestingly, breast invasive carcinoma (BRCA) and prostate adenocarcinoma (PRAD) tumors are predicted to be the least sensitive to trametinib among the 33 TCGA tumor types (Fig. 3a).

Although the median predicted IC_50_ values of trametinib for samples from other tumor types were relatively high, some individual tumor samples were predicted to be as sensitive as the colorectal tumor samples to trametinib, e.g., a few of the PAAD (pancreatic adenocarcinoma) samples. The ability to predict drug sensitivity of individual tumors is important for personalized medicine.

**Fig. 4:**
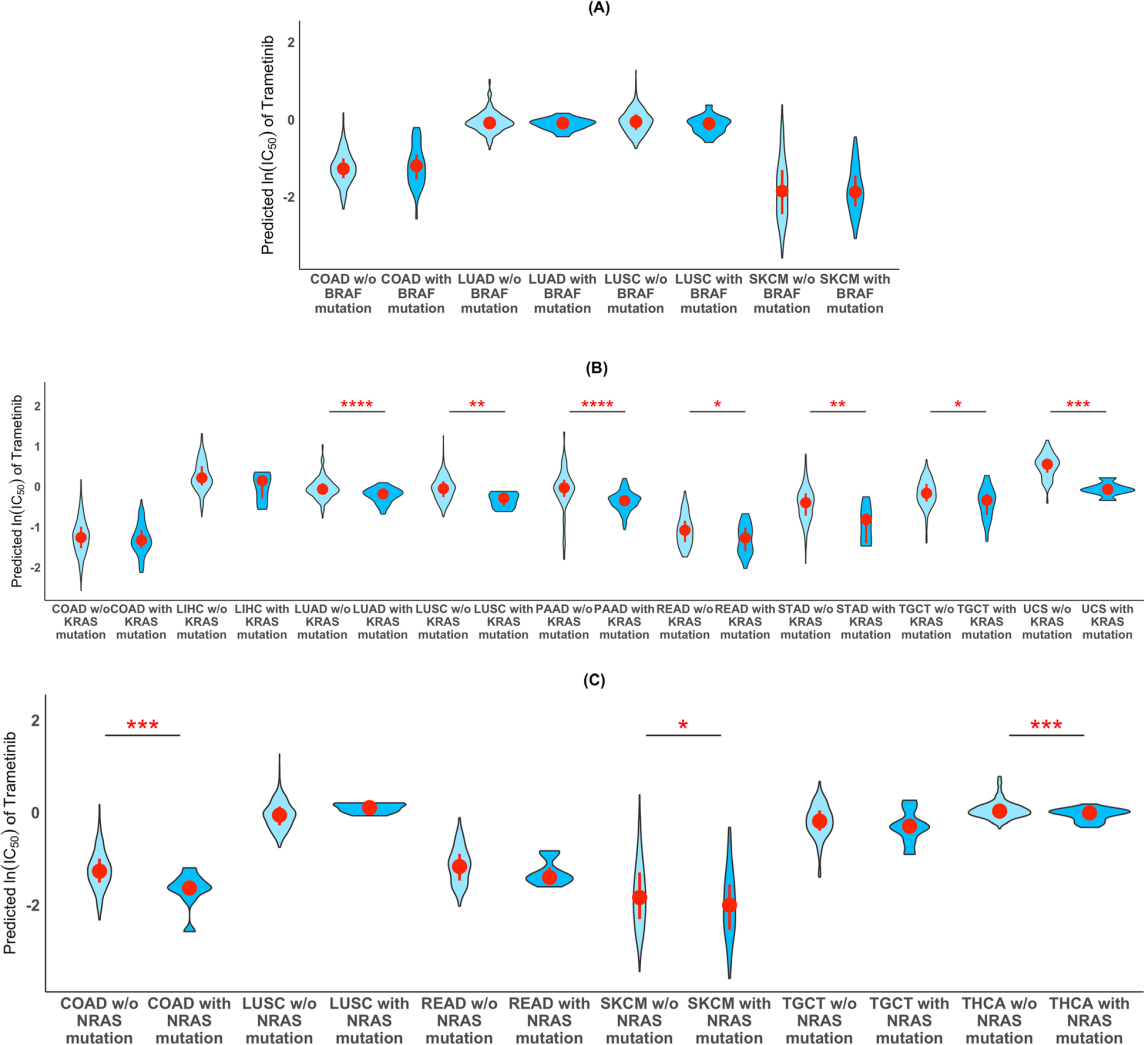
Predicted sensitivity based on mutation status. Violin plots of predicted ln (IC_50_) values of trametinib based on RNA-seq gene expression data from TCGA tumor samples for those with and without mutations in *BRAF* **a**, *KRAS* **b** and *NRAS* **c**. * *p* < 0.05; ** *p* < 0.005; **** *p* < 10^− 6^. Wilcoxon rank-sum test, two-sided

We examined whether the predicted sensitivity of tumor samples to trametinib is correlated with gene mutations. We analyzed all 31 TCGA tumor types (see Methods). For each gene, we divided the TCGA samples for each tumor type into two groups based on whether or not the sample carried mutations in that gene. We considered all genes in the mutation data. To conduct this analysis on a gene and tumor type combination, we required the tumor type to have at least five samples that carried the gene mutation. We then used the Wilcoxon rank-sum test to assess whether predicted sensitivity to trametinib differs between the two groups. Interestingly, we found that *BRAF* mutations were not associated with the predicted sensitivity to trametinib (Fig. 4a). However, among all genes analyzed, we found that *KRAS* is the top-ranked gene whose mutation status was significantly associated with predicted tumor sensitivity to trametinib for the largest number of tumor types (seven of the 31) (Fig. 4b). Similarly, a close related oncogene, *NRAS* whose mutation status was found to significantly associated with the predicted sensitivity to trametinib for three tumor types (Fig. 4c). Specifically, we predicted that patients with either *KRAS* or *NRAS* mutations would be more sensitive to trametinib than those without the mutations. *NRAS* and *KRAS* are upstream molecules of the *BRAF* signaling pathway [50]. Those results suggest that our algorithm selected genes whose transcriptomic profiles are indictive of a role for broader RAS signaling in trametinib sensitivity. Other top-ranked genes whose mutations were found to be associated with predicted sensitivity to trametinib in four of the tumor types include *ADAMTS2*, *ANKRD5*, *MYCBP2*, *TTN*, and *VCAN*.

### Predicting sample-specific IC_50_s for all TCGA and GTEx samples to all 272 drugs

After establishing the potential utility of our concept, we predicted the sensitivities (IC_50_ values) of all TCGA (additional file 6: Table S5) and GTEx samples for the top 272 drugs using our tumor and GTEx data, respectively. Overall, most of the TCGA tumor samples were predicted to be highly sensitive (pan cancer median predicted ln (IC_50_) < 0) to about 35 of the 272 drugs (additional file 5: Table S4**)**. Many of the drugs target DNA/protein synthesis, cell cycle, microtubules, and the mTOR pathway. Most of the drugs were also predicted to be similarly cytotoxic to normal samples from TCGA (additional file 5: Table S4). Those drugs are among the most commonly used chemotherapeutic agents. Unfortunately, they are also associated with high cytotoxicity to normal organs.

### Tumor-to-normal sensitivity

For each of the 272 drugs, we compared the median predicted IC_50_ of the drug for all tumor samples with the median predicted IC_50_ value for all normal samples from the same tumor type from TCGA. We only considered the 14 tumor types (BRCA, COAD, HNSC, KICH, KIRC, KIRP, LIHC, LUAD, LUSC, PRAD, STAD, STES, THCA, and UCEC) with more than 20 normal samples.

**Fig. 5:**
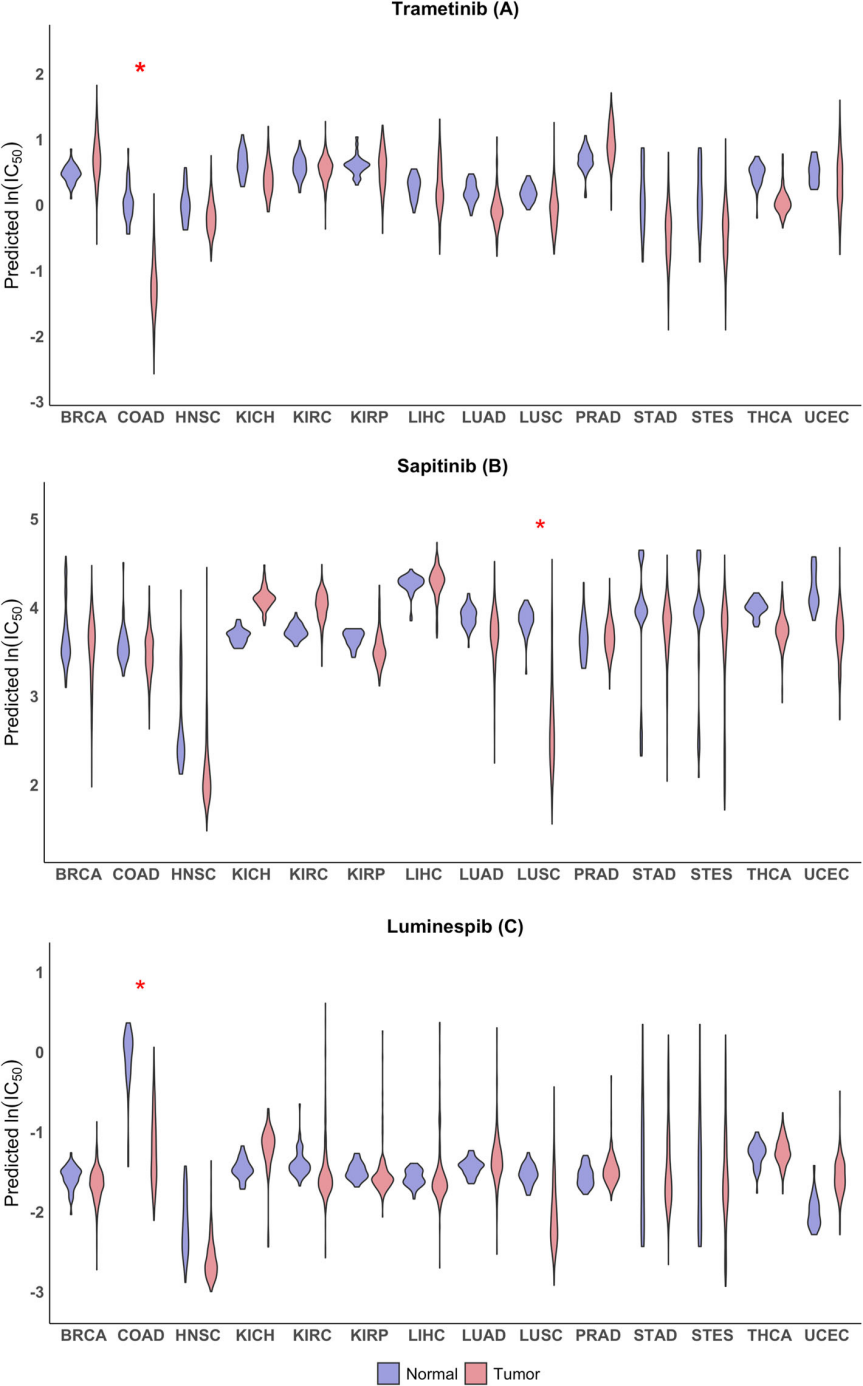
Examples of drugs that are predicted to have high tumor-to-normal sensitivity for some tumor types. Violin plots of predicted IC_50_ values in tumor (red) and normal (blue) tissue for trametinib **a** sapitinib **b** and luminespib **c** that showed the ratio of tumor-to-normal sensitivity exceeding 2.7 (1 logarithmic unit) for at least one of 14 tissue types. The ln (IC_50_) values of the drugs were predicted based on the RNA-seq data of the tumor and normal tissue samples from TCGA. Violin plots for normal and tumor samples from the same tissue type are shown as side-by-side pairs with their TCGA type on the X-axis. See Fig. 2 legend for additional description of the violin plots. Red star (*) indicates the difference between the median of predicted IC_50_ values for normal samples and the median of predicted IC_50_ values for tumor samples is more than one logarithmic unit

We identified eight drugs whose median predicted ln (IC_50_) value for tumor samples was more than one logarithmic unit lower than that for corresponding normal samples in at least one of the 14 tumor types (additional file 4: Fig. S3). One logarithmic unit corresponds to tumor tissue being about 2.7-fold more sensitive than normal tissues. Though we selected drugs based on at least one tumor type having this high ratio of tumor-to-normal sensitivity, for most drugs the high ratio was not limited to a single tumor type. Among the eight drugs, trametinib is an exceptional example for which a drug is predicted to not only be specific for a tumor type (COAD, in this case) but also have high tumor-to-normal sensitivity for only a single tissue type among the 14 tumor-normal pairs (Fig. 5a). Similarly, luminespib (Hsp90 inhibitor) (Fig. 5b) and sapitinib (Erbb inhibitor) (Fig. 5c) are predicted to have high tumor specificity largely for LUSC with high tumor-to-normal sensitivity for the tissue type.

### Tumor-type-specific drugs

For each drug, we compared its median predicted IC_50_ value among samples from one tumor type with the median of the medians of the predicted IC_50_ values from all 33 tumor types. We considered a drug to be specific for a tumor type if the median predicted IC_50_ value for the tumor type is one logarithmic unit (~ 2.7 times) lower than the median of the medians from all tumor types. We identified 109 such drugs (additional file 7: Table S6**)**, most (96) of which were predicted to have lower IC_50_s for either diffuse large B cell lymphoma (DLBC), thymoma (THYM) or both. Interestingly, 12 of the remaining 13 drugs that were predicted not specific for DLBC, THYM or both are kinase inhibitors, consistent with the notion that kinase inhibitors target specific cellular pathways. Eighty-three drugs were predicted to have higher specificity for a unique tumor type; 74 for DLBC, 5 for SKCM (skin cutaneous melanoma), 2 for THYM, and 1 for HNSC (head-neck squamous cell carcinoma) and 1 for KIRP (kidney renal papillary cell carcinoma). Our analysis suggested that the B-Raf proto-oncogene (BRAF) inhibitors (AZ628, Dabrafenib, PLX-4720, and SB590885) are specific for melanoma (additional file 7: Table S6). We also predicted that the four mitogen-activated protein kinase kinase (MEK/ERK) inhibitors (PD0325901, SCH772984, selumetinib, and trametinib) are specific for both colorectal cancer and melanoma. Indeed, clinical trials have demonstrated clinical efficacy of BRAF inhibitors for a portion of melanoma patients harboring activating BRAF mutations [43, 44, 47, 49, 51]. Thus, our predictions are consistent with those human clinical trial results.

**Fig. 6:**
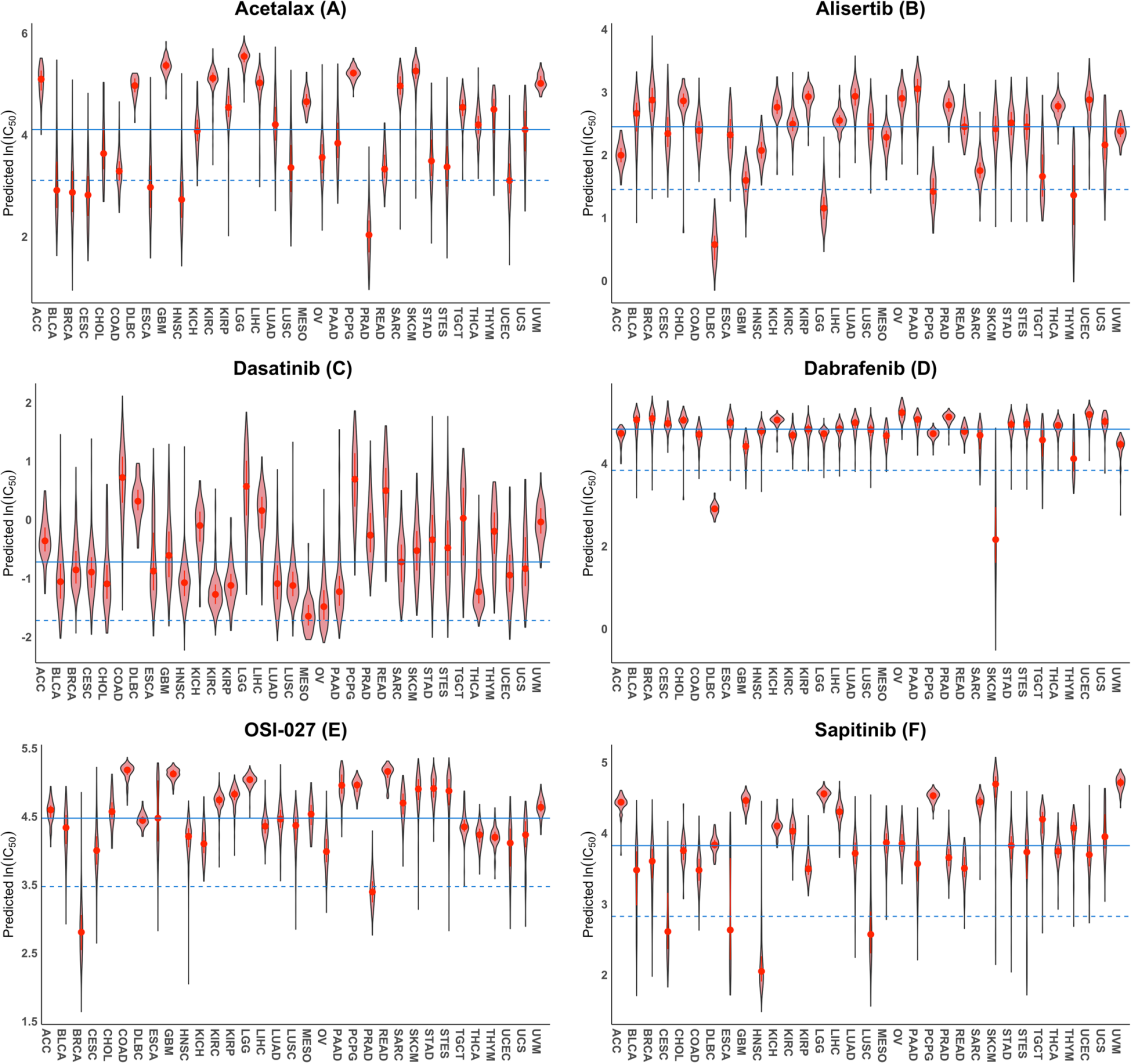
Selected drugs that are predicted to be tumor-type-specific. Violin plots of the predicted ln (IC_50_) values of Acetalax **a**, Alisertib **b**, Dasatinib **c**, Debrafenib **d**, OSI-027 **e**, and Sapitinib **f** for TCGA tumor samples from 33 tumor types. The solid line shows the median of the medians of the predicted IC_50_ values for all 33 tumor types; whereas the dashed line is one logarithmic unit below the solid line. See Fig. 2 legend for additional description of the violin plots

We predicted that acetalax, with unknown mechanism of action, was specific for multiple tumor types including prostate adenocarcinoma (PRAD) and breast invasive carcinoma (BRCA) (Fig. 6a, additional file 7: Table S6). We predicted alisertib was specific for DLBC and lower-grade glioma (LGG) (Fig. 6b). Several tumor types including MESO (mesothelioma) and OV (ovarian serous cystadenocarcinoma) were predicted to be highly sensitive to dasatinib (Fig. 6c). We predicted dabrafenib to be specific for DLBC and SKCM (Fig. 6d). OSI-027 (mTOR inhibitor) showed high specificity to BRCA and PRAD (Fig. 6e). Sapitinib (EGFR/HER2 inhibitor) was specific for HNSC and cervical squamous cell carcinoma and endocervical adenocarcinoma (CESC), esophageal carcinoma (ESCA), and lung squamous cell carcinoma (LUSC) (Fig. 6f).

### Drug sensitivity of breast cancer subtypes

Breast cancers may be classified into subtypes bases gene-expression signatures [52]. To see if subtypes of breast cancer were predicted to show differential sensitivity to any of the 270 drugs, we divided the ~ 1100 TCGA BRCA samples into five subgroups (basal-like, Her2-positive, luminal A, luminal B, and normal-like) based on the PAM50 classification [53, 54]. For each subtype, we compared the median of the predicted IC_50_ values of a drug for the samples of the subtype with the median of the medians of the predicted IC_50_ values for the five subtypes. We focused on drugs for which the difference in the medians exceeded 0.5 logarithmic units (corresponding to a 1.65-fold difference in IC_50_). Among the 270 drugs, seven drugs met this criterion (Table 2). Although OSI-027 did not meet the criterion, we also included it in Table 2 as it is the only drug among the top 272 drugs that showed the highest overall specificity for breast cancer compared to all other TCGA tumor types (Fig. 6e). We showed that Her2-positive breast cancer subtype is predicted to have higher sensitivity to OSI-027 compared to all other four subtypes. We also predicted that basal-like subtype breast cancer has higher sensitivity to five (bleomycin, daporinad, sepantronium bromide, etoposide, and ICL1100013) of the seven drugs, luminal B subtype breast cancer has higher sensitivity to ABT737 and navitoclax, both of which are BCL2 inhibitors.

**Table 2:** Drugs to which breast cancer subtype(s) in TCGA samples were predicted to be sensitive. The lowest predicted ln (IC_50_) values among the subtypes are in bold

Drug			Median Predicted ln (IC_50_) value (Number of samples)				
ID	Name	Target	Basal-like (191)	Her2-pos (82)	Luminal A (567)	Luminal B (219)	Normal-like (41)
1378	Bleomycin	DNA	**2.482**	3.225	3.186	3.371	2.775
1248	Daporinad	NAMPT	**−2.439**	−1.441	−1.665	−1.768	−1.864
268	Sepantronium br.	BIRC5	**−3.622**	−3.388	−2.824	− 3.040	− 2.957
134	Etoposide	TOP2	**1.734**	2.344	2.403	2.369	2.073
1266	ICL1100013	NMT1	**2.443**	3.032	2.988	3.064	2.822
1910	ABT737	BCL2	2.037	2.176	1.500	**1.401**	1.940
1011	Navitoclax	BCL2	1.873	1.951	1.357	**1.188**	1.693
1594	OSI-027	mTOR	2.928	**2.444**	2.829	2.748	2.842

**Fig. 7:**
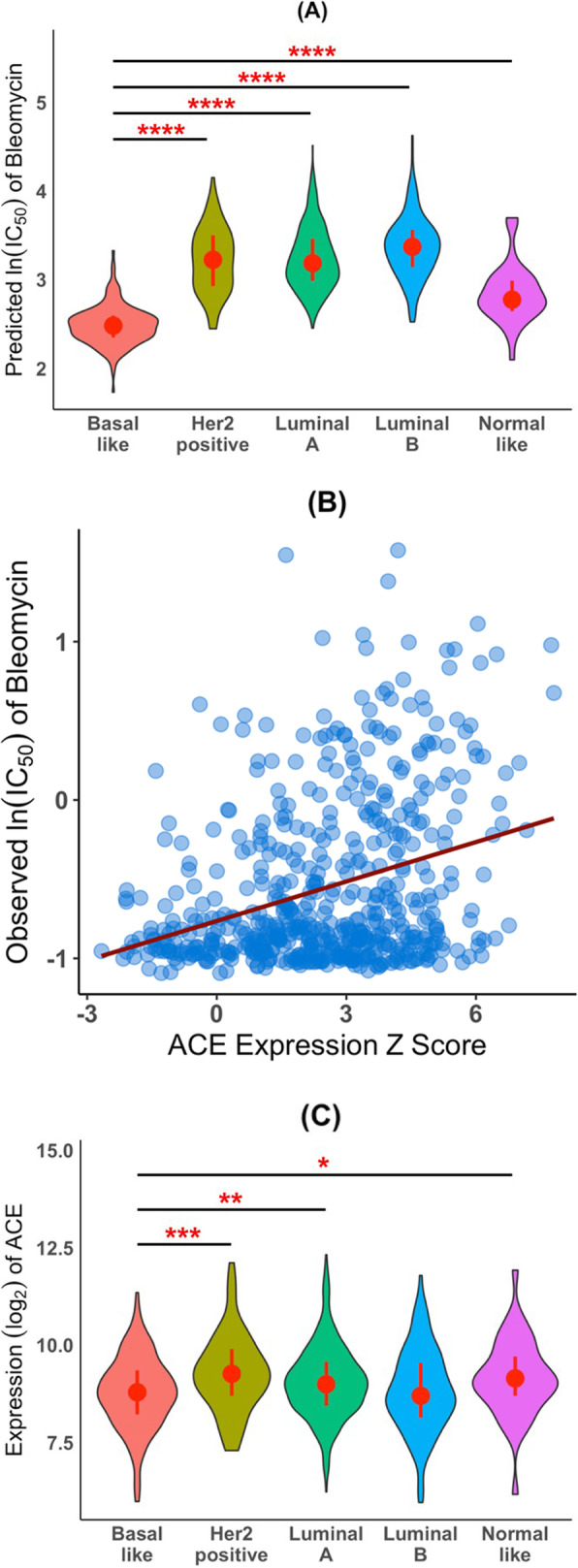
Basal breast tumors are predicted to be more sensitive to bleomycin than luminal A, luminal B or Her2-positive breast tumors and the sensitivity is inversely correlated with *ACE* expression. **a**, Predicted bleomycin sensitivity for the five subtypes of TCGA BRCA samples: violin plots of the predicted ln (IC_50_) values of bleomycin for the five subtypes of breast tumors based on gene expression data and PAM50 classification of TCGA BRCA samples. **b**, *ACE* gene expression in cancer cell lines versus sensitivity to bleomycin: *ACE* expression in the CCLE cancer cell lines was positively correlated with observed ln (IC_50_) values for bleomycin (*ρ*_*s*_ = 0.27, *p*-value = 6.6E-11). The red line is the least-squares regression line. **c**, TCGA breast cancer tumor gene expression data: violin plots of *ACE* expression in TCGA basal-like, Her2-positive, luminal A, luminal B, and normal-like breast tumor samples. * *p* < 0.05; ** *p* < 0.005; **** *p* < 10^− 6^. Wilcoxon rank-sum test, two-sided

Bleomycin is effective for elderly patients with metastatic breast cancer [55]. Bleomycin sulfate followed by electroporation treatment in patients with recurrent in-breast or chest-wall tumors is effective [56]. We predicted that bleomycin has the highest sensitivity for basal-like breast cancer among the three subtypes (Fig. 7a). Interestingly, the most frequently selected gene for predicting sensitivity to bleomycin was *ACE* (additional file 2: Table S2). The *ACE* gene encodes the angiotensin I converting enzyme. Although the exact mechanism of action for bleomycin is unclear, it is thought to inhibit DNA synthesis. *ACE* expression in cancer cell lines was positively correlated with the IC_50_ value of bleomycin in those cell lines (Fig. 7b), suggesting that higher *ACE* expression in cancer cell lines is associated with higher resistance to bleomycin. Although the literature on the relationship between bleomycin and ACE is limited, Day et al. [23] demonstrated that treatment of primary bovine pulmonary artery endothelial cells with bleomycin did increase ACE enzymatic activity and *ACE* mRNA and that the increased ACE expression resulted in fibrosis. Mechanistically, bleomycin activated p42/p44 MAP kinase which in turn up-regulated EGR1, a transcription factor that positively regulates *ACE* expression [23]. Bleomycin-induced ACE overexpression can be inhibited using MEK1/2 inhibitors [23]. Similarly, Li et al. reported that inactivation of ACE alleviated bleomycin-Induced lung Injury [57]. Those studies clearly establish a link between bleomycin and ACE in fibrosis. Interestingly, patients treated with ACE inhibitors have a lower than expected chance of developing cancer [58]. Both fibrosis [59] and MAP kinase activation [60] are associated with tumor progression. Taken together, those results suggest that combination therapy using bleomycin and MEK and/or ACE inhibitors could be beneficial for treating cancers, particularly basal-like type breast cancer.

ACE gene expression in breast basal-like tumor samples was significantly lower than that in all other breast cancer subtypes except luminal B subtype (Fig. 7c). Our algorithm predicted that breast tumors with lower ACE expression are more sensitive to bleomycin than those with higher expression. It is worth to mention that the TCGA tumor samples were taken before any chemotherapy, thus, no induction of ACE expression by bleomycin. Lastly, we would like to emphasize that tumor samples are heterogeneous and ACE gene expression in tumors not only came from cancer cells but also stromal cells. Therefore, the pattern of ACE expression in cancer cell lines may not correlate perfectly well with that in bulk tumor tissues. Our results together with the relationship between bleomycin and *ACE* expression described in the preceding paragraph suggest that patients with basal-like breast tumors would be more sensitive to bleomycin; and if treated with bleomycin, would have lower *ACE* expression, thus, less fibrosis, than similarly treated patients with the luminal subtypes and Her2-positive subtype.

## Discussion

In this work, we began by investigating if a cancer cell line’s transcriptome [4, 5] can predict the IC_50_ of a drug acting on that cell line for each of the 473 GDSC drugs and 1019 cell lines [1–3]. We found that, for about half of the drugs, transcriptomes were reasonably predictive of the sensitivity of the cell lines to those drugs, i.e., that Spearman correlation between predicted and observed IC_50_ values > 0.4.

Among those drugs for which gene expression data can reasonably predict a cancer cell line’s sensitivity, we identified many known drug-gene interactions as well as several novel associations. Our results are consistent with and lend additional support to the notion that the expression levels of *ABCB1* and *SLFN11* are potential biomarkers for cancer cell line sensitivity to multiple drugs [30, 61–63]. Our results also revealed that *SPRY2* expression is positively correlated with the sensitivity of the cancer cell lines to many MEK inhibitors from GDSC, suggesting that *SPRY2* expression may be a predictive biomarker for the effectiveness of MEK kinase inhibitors. We also uncovered that *C19orf33* expression in cancer cell lines is positively correlated (*ρ*_*S*_ ≥ 0.3) with the IC_50_ values of hundreds of chemotherapeutic drugs in those cell lines (additional file 3: Table S3), suggesting that *C19orf33* expression may be a general biomarker for cancer cell line sensitivity to chemotherapeutic agents. Many of the putative biomarkers that we identified in this study may be ‘proxy’ markers for oncomutations. We have no direct evidence of a causal relationship between the expression of the predictive genes and the sensitivity of cell lines to the drugs. Without such evidence, the use transcriptome data to guide clinical practice will remain problematic.

We applied the predictive models learned from the cell line data to both tumor and normal TCGA and normal GTEx RNA-seq data. We used trametinib, a MEK kinase inhibitor [42], as a proof-of-concept exemplar. Based on the GDSC assay data, cancer cell lines from the skin and intestine had the highest sensitivity to trametinib. Trametinib has been shown to be effective in inhibiting the proliferation of BRAFV600E and KRAS mutant cancer cell lines [64]. Clinically, trametinib has been approved for treating cancer patients with the BRAF V600E mutation [43–48]. In clinical practice, MEK inhibitors are used as part of combination treatment to provide potentially clinically relevant activity for colorectal cancer [65]. Conceivably, combination of KRAS inhibitor and MEK inhibitor might be evaluated in clinical trials in the future. Our analyses of the TCGA tumors revealed that trametinib has the highest specificity for melanoma, colorectal cancer, and rectal cancer among all 33 TCGA tumor types, consistent with the clinical application of trametinib. This result prompted us to extend our predictions to the top 272 predictable GDSC drugs, those for which a cancer cell line’s sensitivity can be predicted from transcriptome data.

We found that some of the drugs are highly cytotoxic to all tumors from all tumor types. Those drugs were also predicted to be cytotoxic to normal organ tissues. Unfortunately, those drugs are among the most frequently used chemotherapeutic agents and are associated with side effects. We also identified drugs that show higher specificity towards one or a few tumor types, e.g., ERK, MEK and BRAF inhibitors (AZ628, dabrafenib, PD0325901, PLX-4720, SB590885, SCH772984, and trametinib) for melanoma and colorectal cancer, ERBB/EGFR inhibitors (afatinib and sapitinib) for head-neck squamous cell carcinoma, and BCL2 inhibitors (ABT737, navitoclax and venetoclax) for low grade glioma and glioblastoma multiforme. We further uncovered that OSI-027 was highly specific for breast cancer, especially the Her2-positive subtype breast cancer tumors. Furthermore, our result suggests that *MUCL1* expression may be a surrogate marker for tumor response to OSI-027.

We also predicted that a few drugs not only were tumor-type specific but also induced higher sensitivity in tumor tissue than normal tissue for those tumor types. Those drugs may have better clinical efficacies. Our result suggested that paclitaxel (microtubule inhibitor) (commonly known as taxol) was more specific for breast, lung, and uterine tumors than other tumor types and that those tumors were overall more sensitive to paclitaxel than corresponding normal tissue. Similarly, we predicted that trametinib was specific for melanoma, colorectal tumors and rectal tumors; our analysis also found that those tumors were also more sensitive to trametinib than the normal tissues of the same origins. Sapitinib (ERBB inhibitor) was predicted to have the highest specificity for lung squamous cell carcinoma and was also predicted to be more cytotoxic to lung carcinoma than to normal lung tissue.

We used breast cancer as example to identify tumor subtypes that may be especially sensitive to a drug. For example, we predicted that five drugs (bleomycin, daporinad, sepantronium bromide, etoposide, and ICL1100013) are more specific for basal-like breast cancer subtypes whereas ABT737 and navitoclax sapitinib and afatinib are more specific for the luminal subtypes especially luminal B. We predicted that OSI-027 (mTOR inhibitor) is specific for breast cancer among all 33 TCGA tumor types, especially the Her2-positive breast cancer subtype. Since we have provided the predicted IC_50_ values for all TCGA tumor samples (additional file 7: Table S6), our approach can be easily applied to other tumor types.

It is worth pointing out that OSI-027 was assayed twice (GDSC1 by the Massachusetts General Hospital and GDSC2 by the Sanger) with two different drug IDs (299 for GDSC1 and 1594 for GDSC2). GDSC1 screened 906 cancer cell lines for the drug; whereas GDSC2 screened 265 cancer cell lines. The IC_50_ values of OSI-027 for breast cancer cell lines from GDSC2 are the lowest among all 265 cancer cell lines; however, OSI-027 IC_50_s for breast cancer cell lines from GDSC1 were not among the lowest. Consequently, we predicted that OSI-027 with drug ID 1594, but not drug ID 299, is specific for breast cancer. GDSC recommends using assay data from GDSC2 when available, our results for OSI-027 were based GDSC2 data. However, additional replications are warranted.

There are many challenges associated with relating findings from cell lines to tumors and clinical applications. For example, in vitro assays do not capture organ responses. Although we used the largest collection of cancer cell lines in our predictive models, our models have limitations when applied to datasets that may not be represented by the training data. Nonetheless, translating findings from cell lines to tumors has had some success [5, 8–10, 21, 22].

Our method uses the *k*-nearest neighbor rule to predict drug response of an unknown sample. The predicted value of a sample is taken as the average of the values of its *k*-nearest neighbors. Because of the averaging, the most extreme predicted values, either high or low, usually cannot be as extreme as the corresponding observed values. Therefore, although the correlation between the predicted and observed values can be high, e.g., 0.8, the magnitude of the predicted values is generally pulled in from the extremes; the trend of the predicted values among the samples, however, is usually preserved (Fig. 1). This information should be kept in mind when interpreting the “face value” of predicted values. Lastly, we would like to emphasize that our predictions about sensitivity were solely based on the transcriptomic data; we did not consider information on specific gene mutations. While pan-cancer classification and prediction using transcriptomic data have been successfully applied for identification of biomarkers and of cancer subtypes as well as for prediction of disease progression [66–68], clinical cancer treatment options are often in part guided by oncomutations. Translating results from transcriptomic analyses like ours to clinical practice remains challenging.

## Conclusions

In summary, our predicted drug sensitivity data for all TCGA tumor and normal samples should be a valuable resource to researchers and clinicians. We identified known and novel drug-gene interactions and potential biomarkers for drug effectiveness. Our approach is unique in that we not only predicted drug specificity for tumor types and subtypes, but also drug sensitivity towards normal tissues. We predicted a few drugs to have high specificity for some tumor types compared to all others and high ratios of tumor-to-normal sensitivity. If true, our predictions could have clinical relevance for patients’ care.

## Methods

### Overview of the GA/KNN algorithm

The GA/KNN (genetic algorithm/*k*-nearest neighbors) algorithm combines a genetic algorithm for feature selection and the *k*-nearest neighbor method for classification or prediction [69]. In the present context, the main idea of the GA/KNN algorithm is to use an evolutionary algorithm to select many sets of *d* genes (see below) whose expression levels can accurately predict observed IC_50_ values using the *k*-nearest-neighbors prediction rule. The prediction rule is simple: the predicted IC_50_ value of a sample is defined as the average of the observed IC_50_ values of its *k* nearest neighbor samples (excluding itself) as determined by Euclidean distance in the *d*-dimensional space defined by a gene set. In making predictions for a testing-set sample (see below), we considered only samples within the corresponding training set as potential neighbors.

The GA/KNN algorithm is designed to optimize, either minimize or maximize, an objective function. For prediction, a typical objective function being minimized is the sum of the squared deviations between the observed and predicted IC_50_ values across all training samples (i.e., squared-error loss). The squared-error loss = ${\sum}_{i=1}^N{\left({Obs}_i-{Pred}_i\right)}^2$, where *N* is the number of samples in the training set. The GA/KNN algorithm applied here sought a set of *d* genes to minimize this loss function.

The main parameters for the GA/KNN algorithm were set to be the same for the analyses of all datasets (additional file 1: Table S8**)**. To identify the optimal number of nearest neighbors (*k*) and the “chromosome” length *d*, we systematically evaluated 16 combinations of *k* (*k* = 1, 3, 5, and 7) and *d* (*d* = 10, 20, 30, and 40) (additional file 1: Table S9). Consistent with our earlier findings [69], *k* = 3 and *d* = 30 seemed to provide the near-optimal performance and is computationally efficient.

Because GA/KNN is computationally intensive, we only carried out 100 independent runs for each drug. To see if 100 runs were sufficient, we also analyzed trametinib with 1000 runs. The results from both runs were comparable (additional file 1: Table S10 and additional file 4: Fig. S4).

### Training and cross-validation

For high dimensional data, multiple sets of *d* genes that can deliver similar near-optimal performance. To identify multiple sets of predictive genes, the GA/KNN algorithm uses a Monte Carlo cross-validation procedure [70]. For each drug separately, we randomly partitioned its cell-line data into a training set (90%) and a testing set (10%). We used the training data to identify a set of *d* (*d* = 30) genes whose expression levels were best predictive of the IC_50_ values of samples in the training set using a leave-one-out cross-validation procedure [69]. That set of *d* genes was subsequently used to predict the IC_50_ values of the testing-set samples. The average IC_50_ value of the *k*-nearest (*k* = 3) training neighbors of a testing sample was taken as the predicted IC_50_ value for the testing sample. The above procedure was repeated 100 times independently, each started with a new random partition into training and testing sets. Over the 100 random training-testing partitions for a given drug, each sample would be expected to appear in about 90 training sets and about 10 testing sets. The final predicted value for a training-set sample was the average of the predicted IC_50_ values for that sample over the subset of the 100 independent training-testing partitions in which that sample appeared in a training set; analogously, the final predicted value for a testing-set sample was the average predicted IC_50_ value over the partitions where that sample appeared in a testing set.

### Assessing the importance of individual gene’s expression levels to prediction

Because each training-testing partition provided a set of 30 genes as predictors, we used the frequency with which a gene was selected into the 100 sets of 30 predictor genes as a measure of the importance of that gene in prediction.

### Identifying predictable drugs

We computed both the Pearson (*ρ*_*P*_) and Spearman (*ρ*_*s*_) correlation coefficients between the observed and predicted IC_50_ values for samples in the training and testing sets, respectively. We designated those drugs whose *ρ*_*P*_ and *ρ*_*s*_ values were both greater than or equal to 0.4 (*ρ*_*P*_ ≥ 0.4 and *ρ*_*S*_ ≥ 0.4) for the testing set samples as predictable drugs.

### Predicting IC_50_ values of TCGA tumor samples and GTEx normal tissue samples

In these analyses, we only considered the 272 predictable drugs identified from the cell-line data. For each of the 272 drugs, we repeated the same GA/KNN procedure applied to the cell-line data to both the tumor and the GTEx data. Specifically, for each of the 272 drugs, we randomly partitioned the part of the tumor data from the CCLE cell lines into a training set (90%) and a testing set (10%), repeating the partitioning 100 times, as above. In addition, with each partition, we treated all TCGA samples in the tumor data and the GTEx samples in the GTEx data as additional “testing” samples for prediction.

### Drug sensitivity of cancer cell lines

GDSC screened 397 distinct compounds and 1000 distinct cell lines in two releases: GDSC1 screened 320 compounds in 987 cell lines whereas GDSC2 screened 175 compounds in 809 cell lines. GDSC reports the ln (IC_50_) for each combination of cell line and compound as a measure of the sensitivity of cell viability in that cell line to the compound. We downloaded the ln (IC_50_) data from the GDSC website https://www.cancerrxgene.org/downloads/bulk_download. When combining data from both releases, if IC_50_s for the same cell line and compound were present in both, we kept only the one from GDSC2 as advised by GDSC. In total, 453 drugs were assayed, among which 397 were unique (56 had two different drug IDs). For those with two unique drug IDs, we did not combine them but rather treated each as it were a unique drug as GDSC did on their website. Like GDSC, we refer to the ln (IC_50_) values simply as IC_50_ throughout the manuscript, unless specified otherwise.

### Gene expression of cancer cell lines

CCLE measured gene expression profiles using RNA-seq for 1019 cancer cell lines. We downloaded the gene expression data from the CCLE website (https://portals.broadinstitute.org/ccle/) (CCLE_RNAseq_rsem_genes_tpm_20180929.txt) and converted Ensembl gene IDs into official gene symbols using the annotation file (gencode.v19.genes.v7_model.patched_contigs.gtf). For 111 genes (primarily small nucleolar genes), multiple Ensembl entries corresponded to the same gene symbol; we used the average expression value for those genes. The distribution of the number of cancer cell lines per drug across the 573 drugs is summarized for each cancer type in Table 7A.

### Gene expression of tumor tissue

TCGA makes available RNA-seq gene expression profiles from 771 normal and 10,339 tumor samples encompassing 33 tumor types (additional file 1: Table S7B). We downloaded the RSEM-normalized expression data from the Broad GDAC firehose (https://gdac.broadinstitute.org/). We then log2-transformed those expression values after adding 1 to each.

### Gene expression of normal tissue

GTEx has available RNA-seq gene expression data for normal tissue samples. We downloaded these data (GTEx_Analysis_2017-06-05_v8_RNASeQCv1.1.9_gene_tpm.gct.gz) from the GTEx website https://www.gtexportal.org/home/datasets; we extracted RNA-seq expression data for 5894 tissue samples from 15 major organs (bladder, blood, breast, colon, intestine, kidney, liver, lung, ovary, pancreas, prostate, skin, spleen, stomach, and uterus). We similarly transformed the data as above.

### Combining GDSC drug sensitivity and CCLE gene expression for cancer cell lines

Among the cell lines used by GDSC, we identified all those for which CCLE provided gene expression profiles. Accordingly, for each of the 453 drugs from GDSC, we have CCLE gene expression profiles for a subset of the cell lines with IC_50_s for that drug. Denote the number of such cell lines for drug *D* by *N*_*D*, *CCLE*_ and the number of genes in the expression profile by *G* (same for every drug, *G* = 19163). We created a gene expression data matrix (*G* × *N*_*D*, *CCLE*_) for each drug, with each row indexing a gene and each column indexing a cell line. We also created a corresponding drug-specific vector of IC_50_ values (with length *N*_*D*, *CCLE*_). Here *N*_*D*, *CCLE*_ ranged from 38 to 579 with 25th, 50th, and 75th percentiles of 473, 538, and 553, respectively. For clarity, we refer to these data matrices as “cell-line data”.

### Combining GDSC drug sensitivity and CCLE gene expression with TCGA or GTEx gene expression

We augmented each of the 453 matrices of cell-line data with columns of RNA-seq expression profiles for the tumor samples from the TCGA using the common genes between the two (*G* = 19,163). Thus, we created 400 new expression data matrices (*G* × *N*_*D*, *CCLE* + *TCGA*_), one for each drug. Here *N*_*D*, *CCLE* + *TCGA*_ ranged from 11,129 to 11,670 with 25th, 50th, and 75th percentiles of 11,563, 11,629, and 11,644, respectively. We refer to these data matrices as “tumor data”.

Similarly, we augmented each of the 453 matrices of cell-line data with columns of RNA-seq expression profiles for the normal tissue samples from GTEx using the common genes between the two (*G* = 19163). We created an additional 400 expression data matrices (*G* × *N*_*D*, *CCLE* + *GTEx*_), one for each drug. Here *N*_*D*, *CCLE* + *GTEx*_ ranged from 5932 to 6473 with 25th, 50th, and 75th percentiles of 6367, 6970, and 6447, respectively. We refer to these data matrices as “GTEx data”.

### TCGA tumor mutation data

We downloaded all mutation data from the Broad Institute (https://gdac.broadinstitute.org/) for all 31 TCGA tumor types (Table S7A). We declared a sample to have a gene mutation when any of “nonsense mutation”, “missense mutation”, “frame shift deletion”, “frame shift insertion”, “In frame deletion” or “splice site mutation” was found in the gene for all genes.

### TCGA breast tumor sample clinical data

Hormone status of the TCGA breast invasive carcinoma (BRCA) tumor samples (file name: BRCA.clin.merged.txt) was downloaded from the Broad GDAC firehose (https://gdac.broadinstitute.org/).

### Data integration

When combining data from different sources, it is important that the data are comparable. For this purpose, we computed Z-scores across genes for each cell line from CCLE and each sample from TCGA or GTEx (referred to collectively as “samples”). Thus, each sample has a mean expression of 0 and standard deviation of 1. Let *x*_*i*, *j*_ and *z*_*i*, *j*_ be the log_2_-transformed expression values before and after Z-transformation, respectively, for *j*^*th*^ gene in *i*^*th*^ sample, that is,

$$ {z}_{i,j}=\frac{x_{i,j}-{\overline{x}}_i}{s_i}, $$

where *i* = 1, ⋯*N*_*D*, *CCLE* + *TCGA*_ *or N*_*D*, *CCLE* + *GTEx*_ (depending on the data set) and *j* = *i*, ⋯, *G* and ${\overline{x}}_i$ and *s*_*i*_ are the mean and standard deviation of the expression values for sample *i*.

## Supplementary Information

**Additional file 1: Table S1.** The 10 most predictable drugs. **Table S7.** TCGA tumor types and sample size (A) and summary statistics for the distribution of the number of cancer cell lines per drug across the 573 drugs for each cancer type separately (B). **Table S8.** Main parameters of the GA/KNN algorithm used for the analyses of all datasets. **Table S9.** Training and testing performances for various combinations of *k* and *d*. **Table S10.** Comparison of training and testing performances between two independent runs with 100 and 1000 runs, respectively

**Additional file 2: Table S2.** Drugs for which predictive genes were selected in more than 20 of 100 predictive gene sets

**Additional file 3: Table S3.** The expression level of *C19orf33* in cancer cell lines was positively correlated with the ln (IC_50_) values of more than 100 drugs for those cell lines

**Additional file 4: Figure S1.** Inverse correlation between *SPRY2* expression (Z score) in cancer cell lines and the observed ln (IC_50_) of the six MEK inhibitors for those cell lines. **Figure S2.** *TRPM4* expression (Z score) in cancer cell lines is inversely correlated with observed ln (IC_50_) values of acetalax for the cell lines. **Figure S3.** Drugs that were predicted to have high tumor-to-normal sensitivity for some tumor types. **Figure S4.** Scatter plot of the counts of genes selected into the sets of 30 chromosomes from two independent runs with 100 runs and 1000 runs, respectively

**Additional file 5: Table S4.** Predicted pan-tumor median ln (IC_50_) values for tumor and normal samples

**Additional file 6: Table S5.** Predicted ln (IC_50_) values for all TCGA RNA-seq samples

**Additional file 7: Table S6.** Drugs that were predicted to be specific to tumor type(s)

## Data Availability

The datasets analyzed in this study were downloaded from the following websites: TCGA RNA-seq: https://gdc.cancer.gov/about-data/publications/pancanatlas (filename: EBPlusPlusAdjustPANCAN_IlluminaHiSeq_RNASeqV2.geneExp.tsv); GTEx RNA-seq: https://www.gtexportal.org/home/datasets (filename: GTEx_Analysis_2017-06-05_v8_RNASeQCv1.1.9_gene_tpm.gct.gz); CCLE RNA-seq: https://portals.broadinstitute.org/ccle/ (filename: CCLE_RNAseq_rsem_genes_tpm_20180929.txt.gz); drug sensitivity: https://www.cancerrxgene.org/downloads/bulk_download (filenames: GDSC1_fitted_dose_response_17Jul19.txt & GDSC2_fitted_dose_response_15Oct19.txt); TCGA mutation data: https://gdac.broadinstitute.org/ (filenames: level 3 .maf files). The datasets generated and/or analyzed during the current study are available on https://manticore.niehs.nih.gov/cancerRxTissue

## Abbreviations

TCGA: The Cancer Genome Atlas
GA/KNN: Genetic algorithm and k-nearest neighbors
GDSC: Genomics of Drug Sensitivity in Cancer
CCLE: Cancer cell line encyclopedia

## References

[1] Garnett MJ, Edelman EJ, Heidorn SJ, Greenman CD, Dastur A, Lau KW, Greninger P, Thompson IR, Luo X, Soares J (2012). Systematic identification of genomic markers of drug sensitivity in cancer cells. Nature.

[2] Iorio F, Knijnenburg TA, Vis DJ, Bignell GR, Menden MP, Schubert M, et al. A landscape of Pharmacogenomic interactions in cancer. Cell. 2016;166(3):740–54. 10.1016/j.cell.2016.06.017 PMC4967469 27397505

[3] Yang W, Soares J, Greninger P, Edelman EJ, Lightfoot H, Forbes S, Bindal N, Beare D, Smith JA, Thompson IR et al: Genomics of Drug Sensitivity in Cancer (GDSC): a resource for therapeutic biomarker discovery in cancer cells. Nucleic Acids Res 2013, 41(Database issue):D955–D961. 10.1093/nar/gks1111 PMC3531057 23180760

[4] Ghandi M, Huang FW, Jane-Valbuena J, Kryukov GV, Lo CC, McDonald ER 3rd, et al. Next-generation characterization of the Cancer Cell Line Encyclopedia. Nature. 2019;569(7757):503–8. 10.1038/s41586-019-1186-3 PMC6697103 31068700

[5] Barretina J, Caponigro G, Stransky N, Venkatesan K, Margolin AA, Kim S, et al. The Cancer Cell Line Encyclopedia enables predictive modelling of anticancer drug sensitivity. Nature. 2012;483(7391):603–7. 10.1038/nature11003 PMC3320027 22460905

[6] Seashore-Ludlow B, Rees MG, Cheah JH, Cokol M, Price EV, Coletti ME, et al. Harnessing connectivity in a large-scale small-molecule sensitivity dataset. Cancer Discov. 2015;5(11):1210–23. 10.1158/2159-8290.CD-15-0235 PMC4631646 26482930

[7] Rees MG, Seashore-Ludlow B, Cheah JH, Adams DJ, Price EV, Gill S, et al. Correlating chemical sensitivity and basal gene expression reveals mechanism of action. Nat Chem Biol. 2016;12(2):109–16. 10.1038/nchembio.1986 PMC4718762 26656090

[8] Reinhold WC, Sunshine M, Liu H, Varma S, Kohn KW, Morris J, et al. CellMiner: a web-based suite of genomic and pharmacologic tools to explore transcript and drug patterns in the NCI-60 cell line set. Cancer Res. 2012;72(14):3499–511. 10.1158/0008-5472.CAN-12-1370 PMC3399763 22802077

[9] Reinhold WC, Varma S, Sunshine M, Elloumi F, Ofori-Atta K, Lee S, et al. RNA Sequencing of the NCI-60: Integration into CellMiner and CellMiner CDB. Cancer Res. 2019;79(13):3514–24. 10.1158/0008-5472.CAN-18-2047 PMC6615556 31113817

[10] Rajapakse VN, Luna A, Yamade M, Loman L, Varma S, Sunshine M, et al. CellMinerCDB for integrative cross-database genomics and pharmacogenomics analyses of cancer cell lines. iScience. 2018;10:247–64. 10.1016/j.isci.2018.11.029 PMC6302245 30553813

[11] Nguyen L, Dang CC, Ballester PJ. Systematic assessment of multi-gene predictors of pan-cancer cell line sensitivity to drugs exploiting gene expression data. F1000Res. 2016:5. 10.12688/f1000research.10529.1 PMC5310525 28299173

[12] Wei D, Liu C, Zheng X, Li Y (2019). Comprehensive anticancer drug response prediction based on a simple cell line-drug complex network model. BMC Bioinform.

[13] Suphavilai C, Bertrand D, Nagarajan N. Predicting cancer drug response using a recommender system. Bioinformatics. 2018;34(22):3907–14. 10.1093/bioinformatics/bty452 29868820

[14] Azuaje F, Kaoma T, Jeanty C, Nazarov PV, Muller A, Kim SY, et al. Hub genes in a pan-cancer co-expression network show potential for predicting drug responses. F1000Res. 2018;**7**:1906. 10.12688/f1000research.17149.1 PMC6406180 30881689

[15] Reinhold WC, Varma S, Rajapakse VN, Luna A, Sousa FG, Kohn KW, et al. Using drug response data to identify molecular effectors, and molecular "omic" data to identify candidate drugs in cancer. Hum Genet. 2015;134(1):3–11. 10.1007/s00439-014-1482-9 PMC4282979 25213708

[16] Azuaje F (2017). Computational models for predicting drug responses in cancer research. Brief Bioinform.

[17] Guan NN, Zhao Y, Wang CC, Li JQ, Chen X, Piao X (2019). Anticancer drug response prediction in cell lines using weighted graph regularized matrix factorization. Mol Ther Nucleic Acids.

[18] Guvenc Paltun B, Mamitsuka H, Kaski S. Improving drug response prediction by integrating multiple data sources: matrix factorization, kernel and network-based approaches. Brief Bioinform. 2021;22(1):346–59. 10.1093/bib/bbz153. 10.1093/bib/bbz153 PMC7820853 31838491

[19] Chang Y, Park H, Yang HJ, Lee S, Lee KY, Kim TS, et al. Cancer drug response profile scan (CDRscan): a deep learning model that predicts drug effectiveness from Cancer genomic signature. Sci Rep. 2018;8(1):8857. 10.1038/s41598-018-27214-6 PMC5996063 29891981

[20] Chiu YC, Chen HH, Zhang T, Zhang S, Gorthi A, Wang LJ, et al. Predicting drug response of tumors from integrated genomic profiles by deep neural networks. BMC Med Genet. 2019;12(Suppl 1):18. 10.1186/s12920-018-0460-9 PMC6357352 30704458

[21] Geeleher P, Zhang Z, Wang F, Gruener RF, Nath A, Morrison G, et al. Discovering novel pharmacogenomic biomarkers by imputing drug response in cancer patients from large genomics studies. Genome Res. 2017;27(10):1743–51. 10.1101/gr.221077.117 PMC5630037 28847918

[22] Geeleher P, Cox NJ, Huang RS (2014). Clinical drug response can be predicted using baseline gene expression levels and in vitro drug sensitivity in cell lines. Genome Biol.

[23] Day RM, Yang YZ, Suzuki YJ, Stevens J, Pathi R, Perlmutter A, et al. Bleomycin upregulates gene expression of angiotensin-converting enzyme via mitogen-activated protein kinase and early growth response 1 transcription factor. Am J Resp Cell Mol. 2001;25(5):613–9. 10.1165/ajrcmb.25.5.4521 11713104

[24] Kim JK, Ryll R, Ishizuka Y, Kato S (2000). Identification of cDNAs encoding two novel nuclear proteins, IMUP-1 and IMUP-2, upregulated in SV40-immortalized human fibroblasts. Gene.

[25] Uchiyama S, Itoh H, Naganuma S, Nagaike K, Fukushima T, Tanaka H, et al. Enhanced expression of hepatocyte growth factor activator inhibitor type 2-related small peptide at the invasive front of colon cancers. Gut. 2007;56(2):215–26. 10.1136/gut.2005.084079 PMC1856747 16809422

[26] Kim SJ, An HJ, Kim HJ, Jungs HM, Lee S, Ko JJ, et al. Imup-1 and imup-2 overexpression in endometrial carcinoma in Korean and Japanese populations. Anticancer Res. 2008;28(2A):865–71. 18507030

[27] Ryoo ZY, Jung BK, Lee SR, Kim MO, Kim SH, Kim HJ, et al. Neoplastic transformation and tumorigenesis associated with overexpression of IMUP-1 and IMUP-2 genes in cultured NIH/3T3 mouse fibroblasts. Biochem Biophys Res Commun. 2006;349(3):995–1002. 10.1016/j.bbrc.2006.08.137 16962562

[28] Hodges LM, Markova SM, Chinn LW, Gow JM, Kroetz DL, Klein TE, et al. Very important pharmacogene summary: ABCB1 (MDR1, P-glycoprotein). Pharmacogenet Genomics. 2011;21(3):152–61. 10.1097/FPC.0b013e3283385a1c PMC3098758 20216335

[29] Robey RW, Pluchino KM, Hall MD, Fojo AT, Bates SE, Gottesman MM (2018). Revisiting the role of ABC transporters in multidrug-resistant cancer. Nat Rev Cancer.

[30] Chen KG, Sikic BI (2012). Molecular pathways: regulation and therapeutic implications of multidrug resistance. Clin Cancer Res.

[31] Vassilev LT, Vu BT, Graves B, Carvajal D, Podlaski F, Filipovic Z, et al. In vivo activation of the p53 pathway by small-molecule antagonists of MDM2. Science. 2004;303(5659):844–8. 10.1126/science.1092472 14704432

[32] Gudernova I, Vesela I, Balek L, Buchtova M, Dosedelova H, Kunova M, et al. Multikinase activity of fibroblast growth factor receptor (FGFR) inhibitors SU5402, PD173074, AZD1480, AZD4547 and BGJ398 compromises the use of small chemicals targeting FGFR catalytic activity for therapy of short-stature syndromes. Hum Mol Genet. 2016;25(1):9–23. 10.1093/hmg/ddv441 26494904

[33] Anderson MA, Deng J, Seymour JF, Tam C, Kim SY, Fein J, et al. The BCL2 selective inhibitor venetoclax induces rapid onset apoptosis of CLL cells in patients via a TP53-independent mechanism. Blood. 2016;127(25):3215–24. 10.1182/blood-2016-01-688796 PMC4920022 27069256

[34] Gaspar N, Sharp SY, Pacey S, Jones C, Walton M, Vassal G, et al. Acquired resistance to 17-allylamino-17-demethoxygeldanamycin (17-AAG, tanespimycin) in glioblastoma cells. Cancer Res. 2009;69(5):1966–75. 10.1158/0008-5472.CAN-08-3131 PMC2652695 19244114

[35] Merrill GF, Kurth EJ, Hardie DG, Winder WW (1997). AICA riboside increases AMP-activated protein kinase, fatty acid oxidation, and glucose uptake in rat muscle. Am J Phys.

[36] Boison D (2013). Adenosine kinase: exploitation for therapeutic gain. Pharmacol Rev.

[37] Masoumi-Moghaddam S, Amini A, Morris DL (2014). The developing story of Sprouty and cancer. Cancer Metastasis Rev.

[38] Gross I, Bassit B, Benezra M, Licht JD (2001). Mammalian sprouty proteins inhibit cell growth and differentiation by preventing ras activation. J Biol Chem.

[39] Aytes A, Mitrofanova A, Kinkade CW, Lefebvre C, Lei M, Phelan V, et al. ETV4 promotes metastasis in response to activation of PI3-kinase and Ras signaling in a mouse model of advanced prostate cancer. Proc Natl Acad Sci U S A. 2013;110(37):E3506–15. 10.1073/pnas.1303558110 PMC3773788 23918374

[40] Sagredo AI, Sagredo EA, Pola V, Echeverria C, Andaur R, Michea L, et al. TRPM4 channel is involved in regulating epithelial to mesenchymal transition, migration, and invasion of prostate cancer cell lines. J Cell Physiol. 2019;234(3):2037–50. 10.1002/jcp.27371 30343491

[41] Gao Y, Liao P (2019). TRPM4 channel and cancer. Cancer Lett.

[42] Lugowska I, Kosela-Paterczyk H, Kozak K, Rutkowski P (2015). Trametinib: a MEK inhibitor for management of metastatic melanoma. Onco Targets Ther.

[43] Robert C, Karaszewska B, Schachter J, Rutkowski P, Mackiewicz A, Stroiakovski D, et al. Improved overall survival in melanoma with combined dabrafenib and trametinib. N Engl J Med. 2015;372(1):30–9. 10.1056/NEJMoa1412690 25399551

[44] Long GV, Stroyakovskiy D, Gogas H, Levchenko E, de Braud F, Larkin J, et al. Dabrafenib and trametinib versus dabrafenib and placebo for Val600 BRAF-mutant melanoma: a multicentre, double-blind, phase 3 randomised controlled trial. Lancet. 2015;386(9992):444–51. 10.1016/S0140-6736(15)60898-4 26037941

[45] Grob JJ, Amonkar MM, Karaszewska B, Schachter J, Dummer R, Mackiewicz A, et al. Comparison of dabrafenib and trametinib combination therapy with vemurafenib monotherapy on health-related quality of life in patients with unresectable or metastatic cutaneous BRAF Val600-mutation-positive melanoma (COMBI-v): results of a phase 3, open-label, randomised trial. Lancet Oncol. 2015;16(13):1389–98. 10.1016/S1470-2045(15)00087-X 26433819

[46] Bedard PL, Tabernero J, Janku F, Wainberg ZA, Paz-Ares L, Vansteenkiste J, et al. A phase Ib dose-escalation study of the oral pan-PI3K inhibitor buparlisib (BKM120) in combination with the oral MEK1/2 inhibitor trametinib (GSK1120212) in patients with selected advanced solid tumors. Clin Cancer Res. 2015;21(4):730–8. 10.1158/1078-0432.CCR-14-1814 25500057

[47] Corcoran RB, Atreya CE, Falchook GS, Kwak EL, Ryan DP, Bendell JC, et al. Combined BRAF and MEK inhibition with Dabrafenib and Trametinib in BRAF V600-mutant colorectal cancer. J Clin Oncol. 2015;33(34):4023–31. 10.1200/JCO.2015.63.2471 PMC4669588 26392102

[48] Odogwu L, Mathieu L, Blumenthal G, Larkins E, Goldberg KB, Griffin N, et al. FDA approval summary: Dabrafenib and Trametinib for the treatment of metastatic non-small cell lung cancers harboring BRAF V600E mutations. Oncologist. 2018;23(6):740–5. 10.1634/theoncologist.2017-0642 PMC6067947 29438093

[49] Robert C, Grob JJ, Stroyakovskiy D, Karaszewska B, Hauschild A, Levchenko E, et al. Five-year outcomes with Dabrafenib plus Trametinib in metastatic melanoma. N Engl J Med. 2019;381(7):626–36. 10.1056/NEJMoa1904059 31166680

[50] Moore AR, Rosenberg SC, McCormick F, Malek S (2020). RAS-targeted therapies: is the undruggable drugged?. Nat Rev Drug Discov.

[51] Alcala AM, Flaherty KT (2012). BRAF inhibitors for the treatment of metastatic melanoma: clinical trials and mechanisms of resistance. Clin Cancer Res.

[52] Sotiriou C, Pusztai L (2009). Gene-expression signatures in breast cancer. N Engl J Med.

[53] Wallden B, Storhoff J, Nielsen T, Dowidar N, Schaper C, Ferree S, et al. Development and verification of the PAM50-based Prosigna breast cancer gene signature assay. BMC Med Genet. 2015;8:54. 10.1186/s12920-015-0129-6 PMC4546262 26297356

[54] Chia SK, Bramwell VH, Tu D, Shepherd LE, Jiang S, Vickery T, et al. A 50-gene intrinsic subtype classifier for prognosis and prediction of benefit from adjuvant tamoxifen. Clin Cancer Res. 2012;18(16):4465–72. 10.1158/1078-0432.CCR-12-0286 PMC3743663 22711706

[55] Campana LG, Galuppo S, Valpione S, Brunello A, Ghiotto C, Ongaro A, et al. Bleomycin electrochemotherapy in elderly metastatic breast cancer patients: clinical outcome and management considerations. J Cancer Res Clin Oncol. 2014;140(9):1557–65. 10.1007/s00432-014-1691-6 PMC11823694 24793549

[56] Paramanov V, Tyurin O, Polenkov S, Goldfarb PM (2007). A safety and efficacy study of bleomycin sulfate and electroporation in patients with metastatic or locally recurrent breast cancer. Breast Cancer Res.

[57] Li P, Xiao HD, Xu J, Ong FS, Kwon M, Roman J, et al. Angiotensin-converting enzyme N-terminal inactivation alleviates bleomycin-induced lung injury. Am J Pathol. 2010;177(3):1113–21. 10.2353/ajpath.2010.081127 PMC2928946 20651228

[58] Rosenthal T, Gavras I (2009). Angiotensin inhibition and malignancies: a review. J Hum Hypertens.

[59] Chandler C, Liu T, Buckanovich R, Coffman LG (2019). The double edge sword of fibrosis in cancer. Transl Res.

[60] Dhillon AS, Hagan S, Rath O, Kolch W (2007). MAP kinase signalling pathways in cancer. Oncogene.

[61] Pietanza MC, Waqar SN, Krug LM, Dowlati A, Hann CL, Chiappori A, et al. Randomized, double-blind, phase II study of Temozolomide in combination with either Veliparib or placebo in patients with relapsed-sensitive or refractory small-cell lung cancer. J Clin Oncol. 2018;36(23):2386–94. 10.1200/JCO.2018.77.7672 PMC6085179 29906251

[62] Ballestrero A, Bedognetti D, Ferraioli D, Franceschelli P, Labidi-Galy SI, Leo E, et al. Report on the first SLFN11 monothematic workshop: from function to role as a biomarker in cancer. J Transl Med. 2017;15(1):199. 10.1186/s12967-017-1296-3 PMC5625715 28969705

[63] Vaidyanathan A, Sawers L, Gannon AL, Chakravarty P, Scott AL, Bray SE, et al. ABCB1 (MDR1) induction defines a common resistance mechanism in paclitaxel- and olaparib-resistant ovarian cancer cells. Br J Cancer. 2016;115(4):431–41. 10.1038/bjc.2016.203 PMC4985349 27415012

[64] Lito P, Saborowski A, Yue J, Solomon M, Joseph E, Gadal S, et al. Disruption of CRAF-mediated MEK activation is required for effective MEK inhibition in KRAS mutant tumors. Cancer Cell. 2014;25(5):697–710. 10.1016/j.ccr.2014.03.011 PMC4049532 24746704

[65] Tolcher AW, Peng W, Calvo E (2018). Rational approaches for combination therapy strategies targeting the MAP kinase pathway in solid tumors. Mol Cancer Ther.

[66] Li Y, Kang K, Krahn JM, Croutwater N, Lee K, Umbach DM, et al. A comprehensive genomic pan-cancer classification using The Cancer Genome Atlas gene expression data. BMC Genomics. 2017;18(1):508. 10.1186/s12864-017-3906-0 PMC5496318 28673244

[67] Li YY, Kang K, Krahn JM, Croutwater N, Lee K, Umbach DM, et al. A comprehensive genomic pan-cancer classification using The Cancer Genome Atlas gene expression data. BMC Genomics. 2017:18. 10.1186/s12864-017-3906-0 PMC5496318 28673244

[68] Hoadley KA, Yau C, Hinoue T, Wolf DM, Lazar AJ, Drill E, et al. Cell-of-origin patterns dominate the molecular classification of 10,000 tumors from 33 types of cancer. Cell. 2018;173(2):291–304 e296. 10.1016/j.cell.2018.03.022 PMC5957518 29625048

[69] Li L, Weinberg CR, Darden TA, Pedersen LG (2001). Gene selection for sample classification based on gene expression data: study of sensitivity to choice of parameters of the GA/KNN method. Bioinformatics.

[70] Xu QS, Liang YZ (2001). Monte Carlo cross validation. Chemometr Intell Lab.

