# Supplementary material for: Predicting tumor response to drugs based on gene-expression biomarkers of sensitivity learned from cancer cell lines
Source: BMC Genomics. 2021 Apr 15;22:272. doi: 10.1186/s12864-021-07581-7 (PMC8048084; doi:10.1186/s12864-021-07581-7)
Supplement: Supplementary file 1 — Additional file 1: Table S1. The 10 most predictable drugs. Table S7. TCGA tumor types and sample size (A) and summary statistics for the distribution of the number of cancer cell lines per drug across the 573 drugs for each cancer type separately (B). Table S8. Main parameters of the GA/KNN algorithm used for the analyses of all datasets. Table S9. Training and testing performances for various combinations of k and d. Table S10. Comparison of training and testing performances between two independent runs with 100 and 1000 runs, respectively [file 12864_2021_7581_MOESM1_ESM.docx]

**Table S1**. The 10 most predictable drugs

| GDSC ID | Name | Target | Testing $\rho_{p}$ | Testing $\rho_{s}$ |
| --- | --- | --- | --- | --- |
| 1047 | Nutlin-3a(-) | MDM2 | 0.729 | 0.680 |
| 1909 | Venetoclax | BCL2 | 0.766 | 0.568 |
| 1003 | Camptothecin | TOP1 | 0.654 | 0.664 |
| 1088 | Irinotecan | TOP1 | 0.663 | 0.647 |
| 1190 | Gemcitabine | DNA synthesis | 0.645 | 0.644 |
| 1372 | Trametinib | MEK1, MEK2 | 0.653 | 0.635 |
| 1814 | Nelarabine | DNA synthesis | 0.728 | 0.547 |
| 1563 | EPZ5676 | DOT1L | 0.647 | 0.629 |
| 252 | WZ3105 | NTRK and SRC kinase | 0.650 | 0.620 |
| 1931 | MIRA-1 | TP53 | 0.667 | 0.601 |

**Table S7A**. TCGA Tumor types

| Tumor type | TCGA Tumor Name | No. of samples |
| --- | --- | --- |
| adrenocortical carcinoma | ACC | 79 |
| bladder urothelial carcinoma | BLCA | 408 |
| breast invasive carcinoma | BRCA | 1100 |
| cervical squamous cell carcinoma and endocervical adenocarcinoma | CESC | 306 |
| cholangiocarcinoma | CHOL | 36 |
| colon adenocarcinoma | COAD | 459 |
| esophageal Carcinoma | ESCA | 185 |
| lymphoid neoplasm diffuse large B-cell lymphoma | DLBC | 48 |
| glioblastoma multiforme | GBM | 166 |
| head and neck squamous cell carcinoma | HNSC | 522 |
| kidney chromophobe | KICH | 66 |
| kidney renal clear cell carcinoma | KIRC | 534 |
| kidney renal papillary cell carcinoma | KIRP | 291 |
| brain lower grade glioma | LGG | 530 |
| liver hepatocellular carcinoma | LIHC | 373 |
| lung adenocarcinoma | LUAD | 517 |
| lung squamous cell carcinoma | LUSC | 501 |
| mesothelioma | MESO | 87 |
| ovarian serous cystadenocarcinoma | OV | 307 |
| pancreatic adenocarcinoma | PAAD | 179 |
| pheochromocytoma and paraganglioma | PCPG | 184 |
| prostate adenocarcinoma | PRAD | 498 |
| rectum adenocarcinoma | READ | 95 |
| Sarcoma | SARC | 263 |
| skin cutaneous melanoma | SKCM | 472 |
| stomach Adenocarcinoma | STAD | 415 |
| stomach and Esophageal carcinoma | STES | 600 |
| testicular germ cell tumors | TGCT | 156 |
| thyroid carcinoma | THCA | 509 |
| thymoma | THYM | 120 |
| uterine corpus endometrial carcinoma | UCEC | 177 |
| uterine carcinosarcoma | UCS | 57 |
| uveal melanoma | UVM | 80 |

**Table S7B**. Summary statistics for the distribution of the number of cancer cell lines per drug across the 573 drugs in each cancer type

| Tissue | Min | 1stQu | Median | Mean | 3rdQu | Max |
| --- | --- | --- | --- | --- | --- | --- |
| AUTONOMIC GANGLIA | 0.0 | 8.0 | 9.0 | 9.1 | 11.0 | 11.0 |
| BONE | 0.0 | 10.0 | 11.0 | 10.3 | 11.0 | 11.0 |
| BREAST | 0.0 | 39.0 | 41.0 | 38.1 | 42.0 | 44.0 |
| CENTRAL NERVOUS SYSTEM | 0.0 | 25.0 | 32.0 | 28.5 | 33.0 | 33.0 |
| ENDOMETRIUM | 0.0 | 10.0 | 10.0 | 9.5 | 10.0 | 10.0 |
| HAEMATOPOIETIC AND LYMPHOID TISSUE | 0.0 | 76.0 | 87.5 | 84.4 | 92.0 | 98.0 |
| KIDNEY | 0.0 | 10.0 | 11.0 | 11.2 | 13.0 | 13.0 |
| LARGE INTESTINE | 12.0 | 34.0 | 36.0 | 34.7 | 38.0 | 39.0 |
| LIVER | 0.0 | 11.0 | 11.0 | 10.6 | 12.0 | 12.0 |
| LUNG | 0.0 | 96.8 | 118.0 | 105.9 | 120.2 | 126.0 |
| OESOPHAGUS | 0.0 | 20.0 | 22.0 | 21.2 | 24.0 | 24.0 |
| OVARY | 0.0 | 18.0 | 22.0 | 20.6 | 24.0 | 25.0 |
| PANCREAS | 0.0 | 19.0 | 21.0 | 19.7 | 22.0 | 22.0 |
| PLEURA | 0.0 | 5.0 | 6.0 | 5.5 | 6.0 | 6.0 |
| PROSTATE | 0.0 | 3.0 | 3.0 | 2.8 | 3.0 | 3.0 |
| SALIVARY GLAND | 0.0 | 1.0 | 1.0 | 1.0 | 1.0 | 1.0 |
| SKIN | 0.0 | 20.0 | 27.0 | 23.7 | 28.0 | 30.0 |
| SOFT TISSUE | 0.0 | 12.0 | 12.0 | 12.0 | 13.0 | 14.0 |
| STOMACH | 0.0 | 16.0 | 17.0 | 17.1 | 20.0 | 21.0 |
| THYROID | 0.0 | 7.0 | 8.0 | 7.8 | 9.0 | 9.0 |
| UPPER AERODIGESTIVE TRACT | 0.0 | 10.0 | 12.0 | 11.3 | 14.0 | 14.0 |
| URINARY TRACT | 0.0 | 15.0 | 16.0 | 15.2 | 17.0 | 17.0 |

**Table S8**. Main parameters of the GA/KNN algorithm used for the analyses of all datasets

| Parameter | Value |
| --- | --- |
| population size | 5,000 |
| maximum number of generations | 2,000 |
| chromosome length (*d*) | 30 |
| number of nearest neighbors (*k*) | 3 |
| number of independent GA/KNN runs | 100 |

**Table 9.** Training and testing performances for various combinations of *k* and *d*

| KNN (*k*) | Chromosome length (*d*) | Training | | Testing | |
| --- | --- | --- | --- | --- | --- |
|  |  | Pearson | Spearman | Pearson | Spearman |
| 1 | 20 | 0.926 | 0.911 | 0.624 | 0.611 |
|  | 30 | 0.931 | 0.917 | 0.605 | 0.567 |
|  | 40 | 0.933 | 0.920 | 0.621 | 0.586 |
| 3 | 20 | 0.899 | 0.871 | 0.634 | 0.615 |
|  | 30 | 0.906 | 0.880 | 0.638 | 0.624 |
|  | 40 | 0.912 | 0.890 | 0.620 | 0.589 |
| 5 | 20 | 0.880 | 0.849 | 0.634 | 0.618 |
|  | 30 | 0.890 | 0.864 | 0.625 | 0.606 |
|  | 40 | 0.898 | 0.873 | 0.638 | 0.612 |

**Table S10**. Comparison of training and testing performance between two independent trials with 100 and 1,000 runs, respectively.

| Run | Training | | Testing | |
| --- | --- | --- | --- | --- |
|  | Pearson | Spearman | Pearson | Spearman |
| 100 Runs | 0.908 | 0.886 | 0.627 | 0.608 |
| 1,000 Runs | 0.913 | 0.890 | 0.658 | 0.637 |
