# Supplementary material for: Predicting tumor response to drugs based on gene-expression biomarkers of sensitivity learned from cancer cell lines
Source: BMC Genomics. 2021 Apr 15;22:272. doi: 10.1186/s12864-021-07581-7 (PMC8048084; doi:10.1186/s12864-021-07581-7)
Supplement: Supplementary file 4 — Additional file 4: Figure S1. Inverse correlation between SPRY2 expression (Z score) in cancer cell lines and the observed ln (IC50) of the six MEK inhibitors for those cell lines. Figure S2. TRPM4 expression (Z score) in cancer cell lines is inversely correlated with observed ln (IC50) values of acetalax for the cell lines. Figure S3. Drugs that were predicted to have high tumor-to-normal sensitivity for some tumor types. Figure S4. Scatter plot of the counts of genes selected into the sets of 30 chromosomes from two independent runs with 100 runs and 1000 runs, respectively [file 12864_2021_7581_MOESM4_ESM.docx]

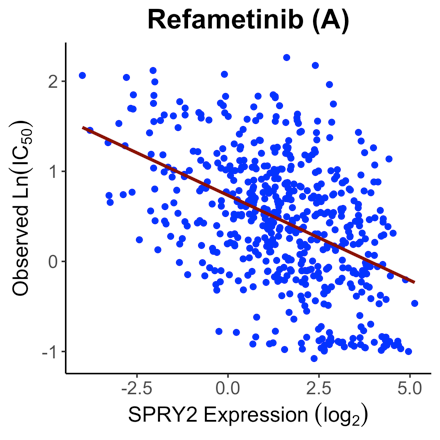

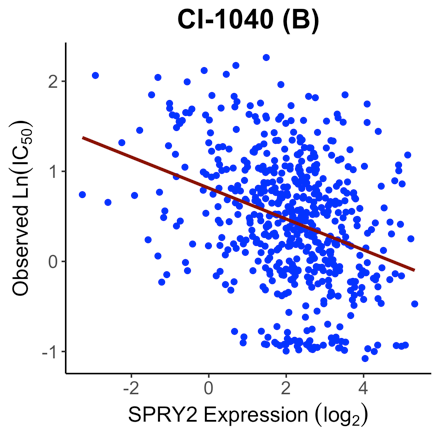

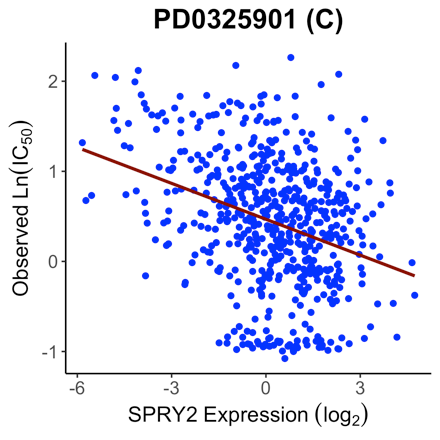

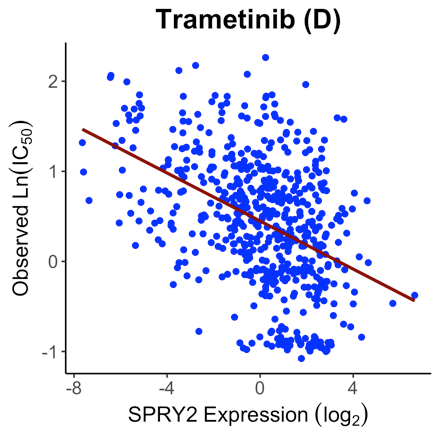

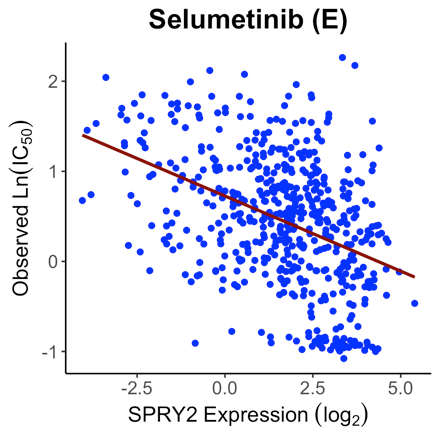

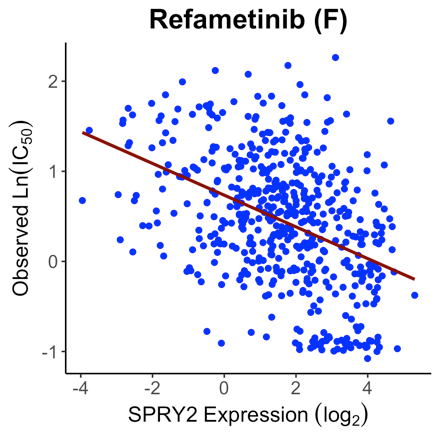


**Figure S1**. Inverse correlation between *SPRY2* expression (Z score) in cancer cell lines and the observed ln(IC_50_) of the six MEK inhibitors for those cell lines. Higher *SPRY2* expression was associated with lower ln(IC_50_) value (more sensitive to these drugs).


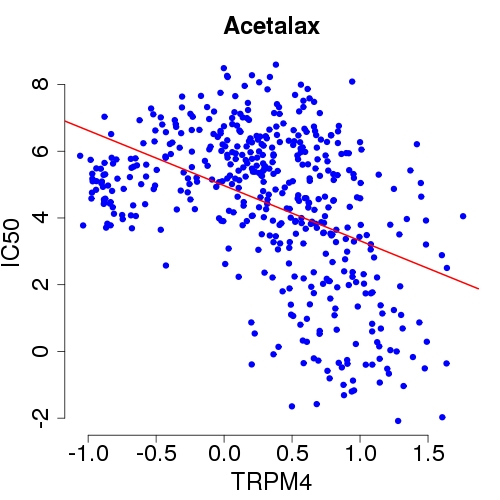


**Figure S2**. *TRPM4* expression (Z score) in cancer cell lines is inversely correlated with observed ln(IC_50_) values of acetalax for the cell lines, indicating high *TRPM4* expression is associated with high sensitivity (low IC_50_) to acetalax.


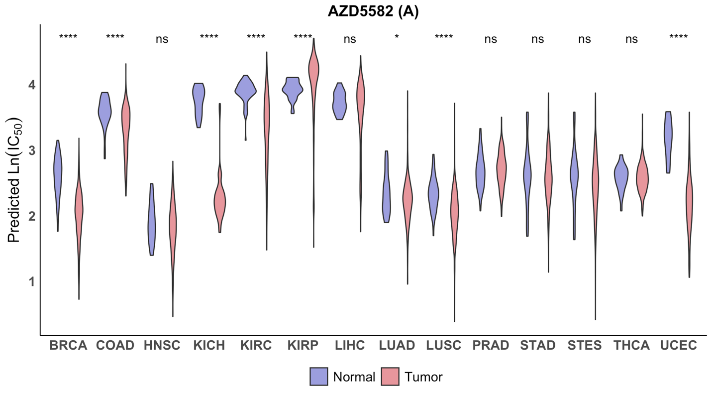

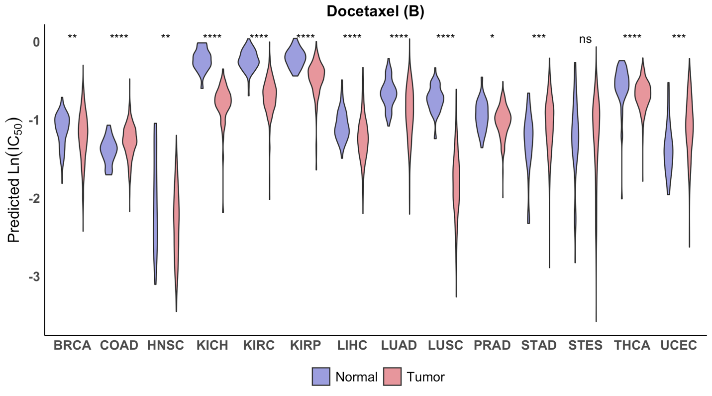

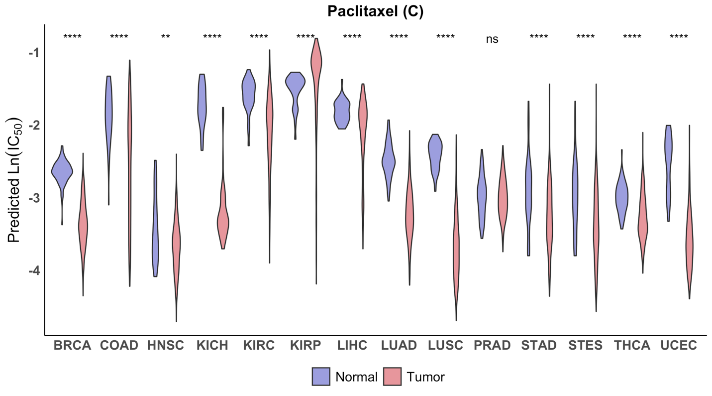

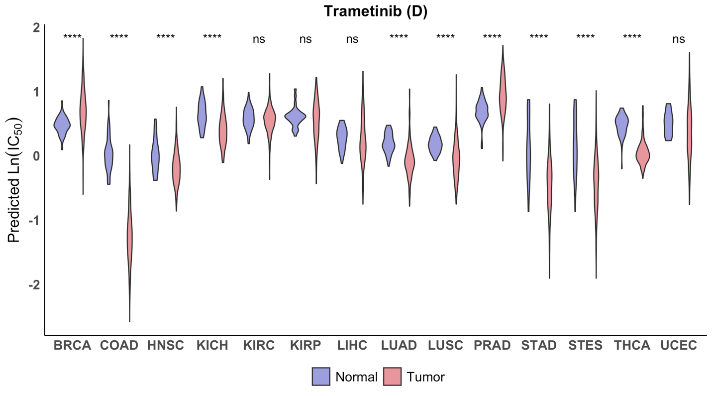

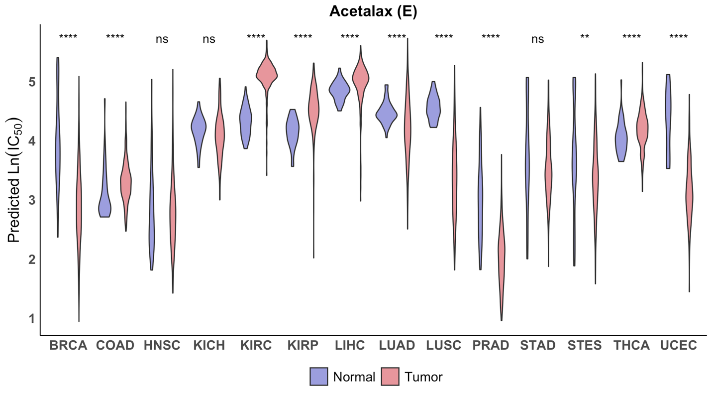

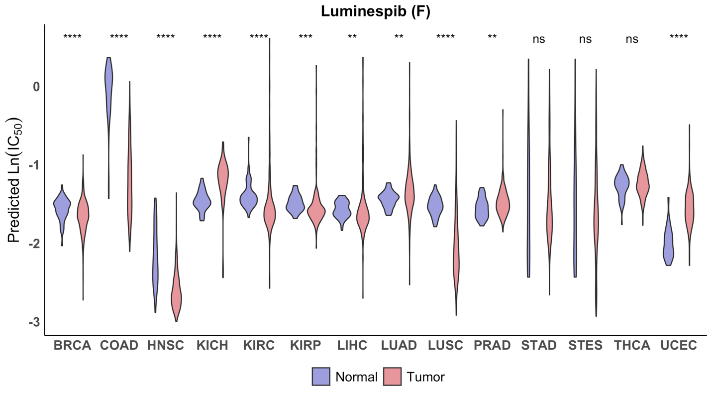

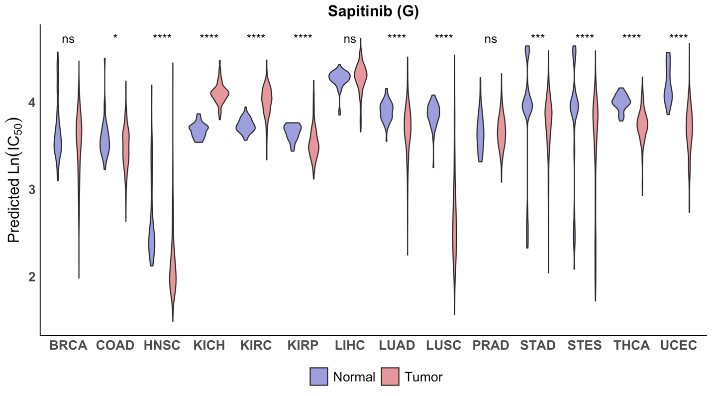

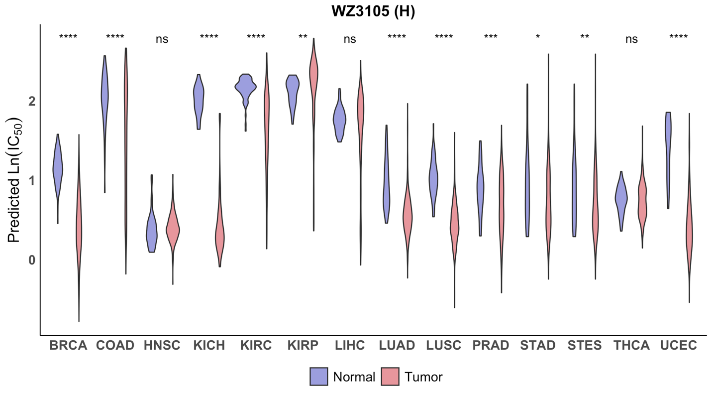


**Figure S3.** Drugs that are predicted to have high tumor-to-normal sensitivity for some tumor types. Violin plots of predicted ln(IC_50_) values in tumor (pink) and normal (blue) tissue for the eight drugs that showed the ratio of tumor-to-normal sensitivity exceeding 2.7 (1 logarithmic unit) for at least one of 14 tissue types. The ln(IC_50_ ) values of the drugs were predicted based on the RNA-seq data of the tumor and normal tissue samples from TCGA. Violin plots for normal and tumor samples from the same tissue type are shown as side-by-side pairs with their TCGA type on the x-axis. See Figure 4 legend for additional description of the violin plots. Statistical significance based on a two-tailed Mann-Whitney-Wilcoxon rank sum test is shown above each pair with ns: p > 0.05, *: p <= 0.05, **: p <= 0.01, ***: p <= 0.001, ****: p <= 0.0001.


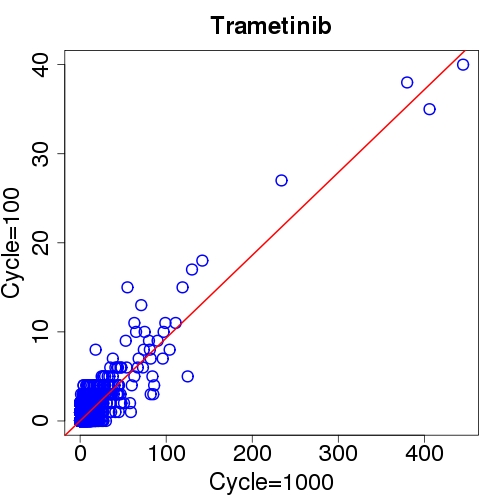


**Figure S4**. Scatter plot of the counts of genes selected into the sets of 30 chromosomes from two independent runs with 100 runs and 1,000 runs, respectively.
